# Better models, better treatment? a systematic review of current three dimensional (3D) *in vitro* models for implant-associated infections

**DOI:** 10.3389/fbioe.2025.1569211

**Published:** 2025-04-25

**Authors:** Neele Brümmer, Katharina Doll-Nikutta, Patrik Schadzek, Carina Mikolai, Andreas Kampmann, Dagmar Wirth, Andrea Hoffmann, Philipp-Cornelius Pott, Oliver Karras, Sören Auer, Meike Stiesch

**Affiliations:** ^1^ Hannover Medical School, Clinic of Prosthetic Dentistry and Biomedical Materials Research, Hannover, Germany; ^2^ Lower Saxony Center for Biomedical Engineering, Implant Research and Development (NIFE), Hannover, Germany; ^3^ Hannover Medical School, DIAKOVERE Annastift, Department of Orthopedic Surgery, Hannover, Germany; ^4^ Hannover Medical School, Clinic for Cranio-Maxillo-Facial Surgery, Hannover, Germany; ^5^ Helmholtz Centre for Infection Research, Braunschweig, Germany; ^6^ Hannover Medical School, Department of Experimental Hematology, Hannover, Germany; ^7^ TIB - Leibniz Information Centre for Science and Technology, Hannover, Germany

**Keywords:** 3D model, *in vitro*, implant, infection, tissue engineering

## Abstract

**Introduction:**

Understanding the biology of implant-associated infections is essential in order to provide adequate detection, prevention and therapeutic strategies. Advanced 3D in vitro models offer valuable insights into the complex interactions between cells and bacteria in the presence of implant materials. This review aims to give a comprehensive overview of current 3D in vitro models that mimic implant-associated infections.

**Methods:**

The structured literature search initially identified 258 publications, seven of which fitted the inclusion criteria.

**Results:**

The included 3D models were established either to mimic the in vivo situation (organotypic model) or to investigate future implant materials. In three studies, organotypic models for dental implants were created and one study described an organotypic model containing immune cells. In the remaining three studies, biomaterials for constructing future orthopedic implants were developed and tested. All authors included specific cells and bacteria suitable for the respective implants. The dental implant models used fibroblasts and keratinocytes; the orthopedic implant models used stem cells and fibroblast-like cells; the model containing immune cells incorporated co-cultivation of fibroblasts and THP-1 derived macrophages. For bacterial challenge, most authors used Gram positive bacteria, but three studies employed Gram negative bacterial species. A wide variety of analytical methods of different complexity were applied after co-culture of cells and bacteria and between one and five different methods were used.

**Discussion:**

All models could be employed to provide answers to specific scientific questions regarding implant-associated infections. Nonetheless, this review reveals the limitations of current 3D models for the investigation of implant-associated infections and highlights the opportunities for further development in this scientific field.

## 1 Introduction

Implants are widely used across various medical disciplines, primarily to replace terminally damaged or impaired parts of the human body, such as dental implants for teeth and orthopedic prostheses for joints. Despite their significant success and widespread application in numerous fields (Kapadia et al., 2016; Adler et al., 2020), implants remain susceptible to various complications associated with mechanical or biological problems. Biological complications are often related to bacterial infections, and may cause severe illness and necessitate intensive therapy. These challenges are further exacerbated by the increasing prevalence of antibiotic resistance (Kim et al., 2020; Premkumar et al., 2021). Infections are principally of importance for prosthetic and dental implants and their rates vary significantly with the implant type. Prosthetic joint infections of total hip and total knee arthroplasties have an incidence of 0.8%–3% (Kim et al., 2020; Premkumar et al., 2021; Mansour et al., 2024), but lead to more than 25% of all revision surgeries (Kapadia et al., 2016). Bacterial infections of dental implants associated with a loss of alveolar bone, known as “peri-implantitis”, have a prevalence of about 21% after 10 years (Derks and Tomasi, 2015; Dreyer et al., 2018). Implant-associated infections in general are characterized by bacterial biofilms formed on biomaterial surfaces that hinder or destroy the physiological tissue integration of the implant (Dreyer et al., 2018). Within biofilms, microorganisms form complex symbiotic relationships embedded in a matrix that increases the tolerance to chemical and mechanical attacks (Kapadia et al., 2016). As a consequence, therapy of biofilm-caused infections still remains a challenge.

One strategy to reduce the number of implant-associated infections is to develop novel implant materials for the prevention of biofilm formation and of new implant-related methods for early detection of implant-associated infections. These could assure early treatment at a prognostically favorable state before the infection is too advanced.

In recent decades, cytocompatibility and antibacterial behavior of such new implant systems have been tested through classical two-dimensional (2D) techniques of cell and bacteria culture. These were either monocultures of relevant cells and bacterial species or co-cultures of different cell types or cells and bacteria (Moriarty et al., 2014). These 2D *in vitro* models serve to answer a wide range of immediate biological questions in short time and at low costs, but they also greatly simplify the complexity of a real implant environment. For example, they lack physiological cell-to-cell contacts and differ from real tissue in cell morphology and cell behavior (Yamada and Cukierman, 2007). Thus, they cannot lead to a full understanding of the multifaceted interactions between cells, matrix, microorganisms and implant materials (Farshidfar et al., 2023). In the regular sequence, relatively simple 2D models would be followed by complex *in vivo* animal experiments, which are prerequisite for clinical studies. Simplification of complex biological systems is not an issue in animal experiments but however, they are restricted by ethical problems and are subject to constantly increasing regulatory requirements, so that concerns about this gold standard in biomaterial science are constantly growing. Moreover, animal studies are often expensive, laborious and the transferability of results to humans might be limited (Yamada and Cukierman, 2007; Moriarty et al., 2014).

Complex 3D *in vitro* models have emerged in recent years to close the gap between 2D models and *in vivo* experiments. In comparison to 2D models, 3D *in vitro* models include physiological cell-to-cell contacts and provide an alternative approach to investigate the interaction between tissue cells, implant materials and pathogenic microorganisms. In comparison to *in vivo* models, these 3D models offer the advantages that human cells can be used and that 3D models allow the mechanisms of pathogenesis to be elucidated at the cellular and molecular levels. 3D models can also contribute to the goals of the 3R Principle (Replacement, Reduction, and Refinement of *in vivo* experiments). The application of 3D models before *in vivo* experiments can reduce the number of needed animals by refining the scientific questions at an earlier stage. In case of negative results after 3D *in vitro* studies, that were not found earlier in 2D models, the *in vivo* study of these particular conditions might not be reasonable after all. Those benefits of 3D models can prospectively lead to lower cost and time requirements for the development and legalization of diagnostic methods and treatment options. For this purpose, different methods to create artificial 3D tissues have been developed. One approach is to use harvested tissue from human donors and further cultivate it *in vitro*. This approach enables biological testing under real conditions, but does not allow long term observation because of limited culture duration (Yamada and Cukierman, 2007). Scaffold-based models use a 3D structure made from different materials (ranging from soft structures like hydrogels up to rigid scaffolds like ß-TCP) to guide tissue-like cellular growth (AlFatlawi et al., 2023; Shayya et al., 2024). The models can be further structured using transwell systems with semipermeable membranes to separate different cell types but allow interaction via soluble substances (Shayya et al., 2024). 3D *in vitro* models that investigate cell-cell interactions under physiological and pathological conditions or test new treatment options have recently been reviewed (Yamada and Cukierman, 2007; AlFatlawi et al., 2023; Farshidfar et al., 2023; Eijkel et al., 2024; Shayya et al., 2024). The aim of the present systematic review is, thus, to provide a comprehensive overview on 3D *in vitro* systems that are specifically dedicated to implant-associated infections. By summarizing and comparing their respective designs - including cell types, implant materials, bacterial strains and culture conditions, as well as the analytical methods used - the current possibilities and limitations will be highlighted.

## 2 Materials and methods

### 2.1 Protocol development

For this systematic review, the Preferred Reporting Items for Systematic Review and Meta-Analyses (PRISMA) guidelines were followed (Page et al., 2021). The systematic literature search was based on the following PICOS question:

“Which *in vitro* models dedicated to investigate implant-associated infections exist that include relevant cells in a 3D culture setup, relevant bacteria for implant-associated infections and implant materials? How do these models resemble and differ from native tissues?”

Accordingly, the PICOS were:

Population: *In vitro* models for implant-associated bacterial infections including cells (at least one relevant cell type for the investigated tissue) cultured in a 3D setup, at least one bacterial strain relevant for infection and a biomaterial used for implants.

Intervention: N/A.

Comparison: Native human tissue with implant-associated infection.

Outcome: Overview of used cell types, bacterial species and biomaterials; similarities and differences from human tissue.

Studies: *In vitro* experiments.

### 2.2 Search strategy

Based on the defined PICOS, the search terms displayed in Table 1 were built for three databases (Pubmed, Web of Science and Scopus) that were searched individually. Additionally, a manual search was performed of the references in the included publications.

**TABLE 1:** Terms used for systematic literature search in Pubmed, Web of Science and Scopus.

Database	Search term
Pubmed	(“Cell Culture Techniques, Three Dimensional” [Mesh] OR “Cell Culture*” [tiab] OR “Coculture Techniques” [Mesh] OR “Coculture Techniques” [tiab] OR “Co-culture*” [tiab] OR “Coculture*” [tiab] OR “Co culture*” [tiab] “Cocultivation*” [tiab] OR “Microphysiological Systems” [Mesh] OR “organotypic model*” [tiab] OR “organotypic culture*” [tiab] OR “organotypic cell culture*” [tiab]OR “Organoids” [Mesh] OR “organoid*” [tiab] OR “3D model*” [tiab] OR “three-dimensional model*” [tiab] OR “organotypic mucosa” [tiab]) AND (“Cells” [Mesh] OR “cell*” [tiab] OR “Osteoblasts” [Mesh] OR “Osteoblast*” [tiab] OR “Fibroblasts” [Mesh] OR “Fibroblast*” [tiab] OR “Epithelial Cells” [Mesh] OR “Epithelial cell*” [tiab] OR “tissue” [tiab] OR “mucosa” [tiab] OR “bone” [tiab]) AND (“Bacteria” [Mesh] OR “bacteria*” [tiab] OR “Biofilms” [Mesh] OR “biofilm*” [tiab] OR “*Staphylococcus aureus*” [Mesh] OR “*Staphylococcus aureus*” [tiab] OR “*Staphylococcus* epidermidis” [Mesh] OR “*Staphylococcus* epidermidis” [tiab] OR “Porphyromonas gingivalis” [Mesh] OR “Porphyromonas gingivalis” [tiab] OR “*Fusobacterium* nucleatum” [Mesh] OR “*Fusobacterium* nucleatum” [tiab] OR “*Streptococcus*” [Mesh] OR “*Streptococcus*” [tiab]) AND (“Dental Implants” [Mesh] OR “dental implant*” [tiab] OR “Prostheses and Implants” [Mesh] OR “prosthesis*” [tiab] OR “prostheses*“ [tiab] OR “endoprosthesis*“ [tiab] OR “endoprostheses*“ [tiab] OR “prosthetic*“ [tiab] OR “implant*” [tiab] OR “Titanium” [Mesh] OR “titanium*” [tiab] OR “Ceramics” [Mesh] OR “ceramic*” [tiab] OR “zirconia*” [tiab] OR “zirconium dioxide*” [tiab] OR “Chromium Alloys” [Mesh] OR “chromium alloy*” [tiab] OR “cobalt-chromium alloy*” [tiab] OR “cobalt chromium alloy*” [tiab])
Web of Science	(TS=((“Cell Culture Techniques, Three Dimensional” OR “Cell Culture” OR “Coculture Techniques” OR “Co-culture” OR “Coculture” OR “Co culture” “Cocultivation” OR “organotypic model” OR “organotypic culture” OR “organotypic cell culture” OR “Organoid” OR “3D model” OR “three-dimensional model” OR “organotypic mucosa”) AND (“Cells” OR “Osteoblast” OR “Fibroblast” OR “Epithelial Cell” OR “tissue” OR “mucosa” OR “bone”) AND (“Bacteria” OR “Biofilm” OR “*Staphylococcus aureus*” OR “*Staphylococcus* epidermidis” OR “Porphyromonas gingivalis” OR “*Fusobacterium* nucleatum” OR “*Streptococcus*”) AND (“Dental Implants” OR “Prostheses and Implants” OR “prosthesis” OR “prostheses” OR “endoprosthesis” OR “endoprostheses” OR “prosthetic” OR “implant” OR “Titanium” OR “Ceramics” OR “zirconia” OR “zirconium dioxide” OR “Chromium Alloys” OR “cobalt-chromium alloy” OR “cobalt chromium alloy”)))
Scopus	(“three dimensional cell culture” OR “Co-culture*” OR “Coculture” OR “Co Culture” OR “Cocultivation” OR “organotypic model*” OR “organotypic Pre/2 culture*” OR “Organoid*” OR “3D W/2 model” OR “Three-dimensional W/2 model*” OR “organotypic mucosa”) AND (“Cell*” OR “Osteoblast*” OR “Fibroblast*” OR “Epithelial Pre/2 cell*” OR “tissue” OR “mucosa” OR “bone”) AND (“bacteria*” OR “biofilm*” OR “*Staphylococcus aureus*” OR “*S. aureus*” OR “*Staphylococcus* epidermidis” OR “S. epidermidis” OR “Porphyromonas gingivalis” OR “*Fusobacterium* nucleatum” OR “*Streptococcus*”) AND (“dental implant*” OR “prosthes?s*” OR “endoprosthes?s*” OR “prosthetic*” OR “implant*” OR “titanium*” OR “ceramic*” OR “zirconia*” OR “zirconium dioxide*” OR “chromium alloy*” OR “cobalt-chromium alloy*” OR “cobalt chromium alloy*”)

### 2.3 Eligibility criteria and search process

The literature search was conducted in May 2024 and revealed studies published between 1988 and 2024 (May). Two independent scientists (NB and KDN) sorted the publications according to the eligibility criteria (Table 2). In the event of conflicts, a third scientist (PS) was consulted and consensus was found. Data extraction was finished in July 2024.

**TABLE 2:** Eligibility criteria.

Inclusion criteria	Exclusion criteria
Studies of 3D *in vitro* models including the three components, cells cultured in 3D (relevant to investigated tissue), bacteria (relevant to implant-associated infection) and an implant material	*In vivo* studies
	2-dimensional cell culture models
	Studies using cells that are not relevant for the investigated tissue
English or German language	Studies using bacteria that are not relevant for implant-associated infections
	Studies using biomaterials not for implant usage
	Reviews

### 2.4 Data extraction and statistical analyses

From the included studies, the following data was extracted: model setup including cell type and culture condition, bacterial strain and culture condition, implant material and 3D model build-up as well as outcome analysis, including cell culture-based methods, molecular methods, microscopy, histology and combinations thereof. Due to the low number of studies fulfilling inclusion criteria, analysis was performed on a qualitative basis and no meta-analysis was performed. The extracted data is organized and published in the Open Research Knowledge Graph (ORKG) (Auer et al., 2025) as an ORKG comparison (Brümmer et al., 2025). In this way, a detailed and interactive overview of the organized data can be provided to ensure a better accessibility and sustainability (Karras et al., 2024).

### 2.5 Risk of bias assessment

Two reviewers (NB and PS) performed the risk of bias assessment in duplicate and individually. In case of conflicts, a third and fourth scientist (KDN and CM) were consulted and consensus was found. There are no established criteria for evaluation of *in vitro* studies. Therefore, the risk of bias was evaluated according to the articles’ description of the following parameters: Clearly stated aims/objectives, sample size rationale depending on the stated aims, standardization in model production, implant material characterization, evaluation of cell morphology and viability, evaluation of bacterial viability, observer blinding and adequate statistical analysis. Only those experimental methods were taken into account that were performed after co-culture of cells and bacteria in the presence of the implant material. If the domains were reported, the study was presented with score 1, or score 0 if data were missing. Studies that scored less than three domains were rated as having a “high” risk of bias, “moderate” if scored four to six and “low” if they scored seven to nine.

## 3 Results

### 3.1 Search results

**FIGURE 1:**
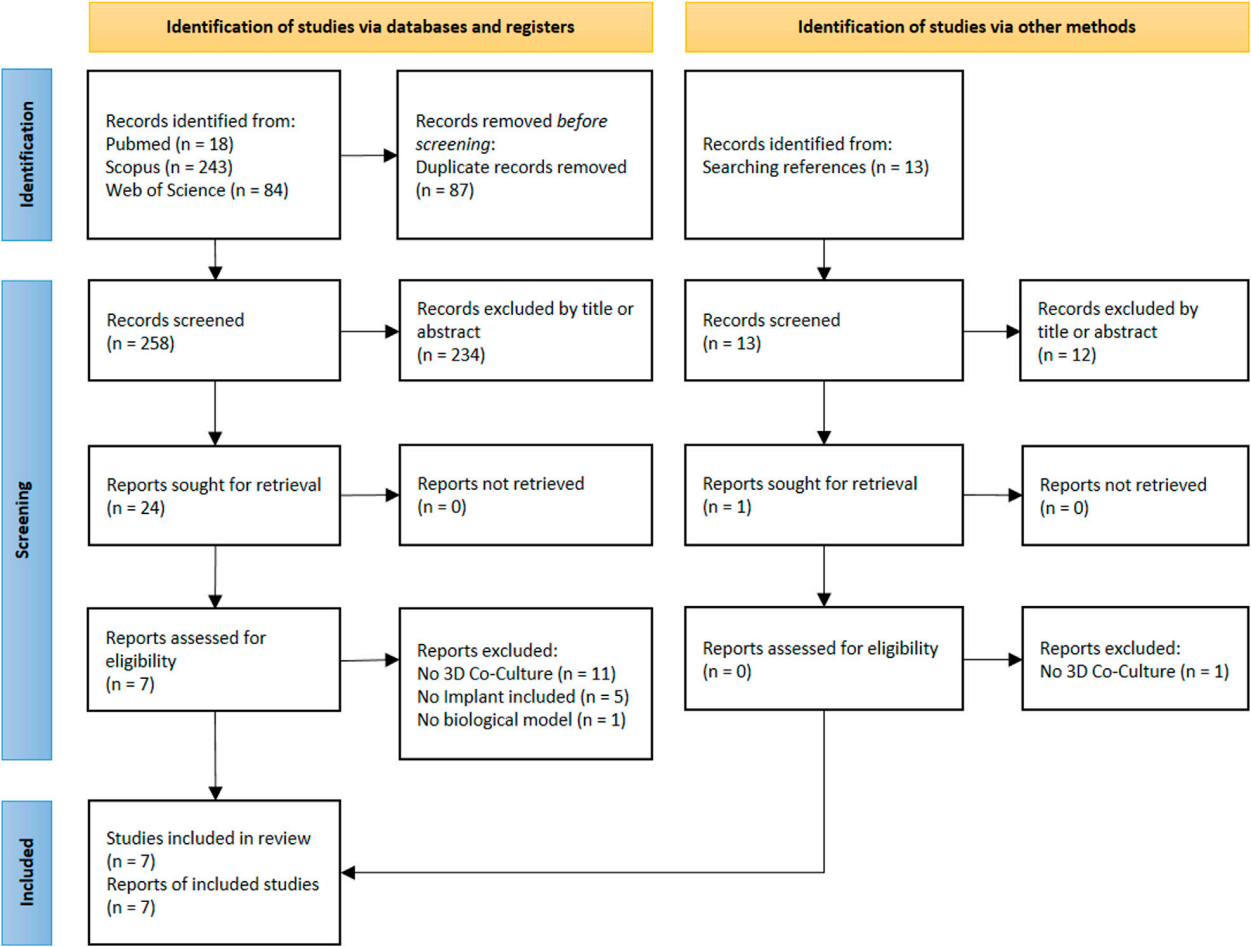
PRISMA flowchart.

The search results are illustrated in the PRISMA flow chart in Figure 1. The initial search in three databases revealed 258 records after duplicate removal. After screening of title and abstract, 24 publications were further considered. Amongst them, seven publications, published between 2016 and 2024, were finally included in this review. An additional search of the publication’s references did not lead to additional inclusions.

### 3.2 *In vitro* model setups

In the seven included studies, different implant systems and tissues were modeled. Dental implant-associated infections were investigated in three studies (Ingendoh-Tsakmakidis et al., 2019; Ren et al., 2019; Mikolai et al., 2020), with the models of Ingendoh-Tsakmakidis et al. (2019) and Mikolai et al. (2020), which were identical but applied to different scientific questions. One study described a 3D model for cartilage repair (Bonifacio et al., 2020) and two models were dedicated to investigate possible methods for the treatment or prevention of osteomyelitis (Mohiti-Asli et al., 2016) or osseous defects (Jia et al., 2016). One model was developed as an immunocompetent model that was adaptable to different tissues and implant materials (Murkar et al., 2024).

**FIGURE 2:**
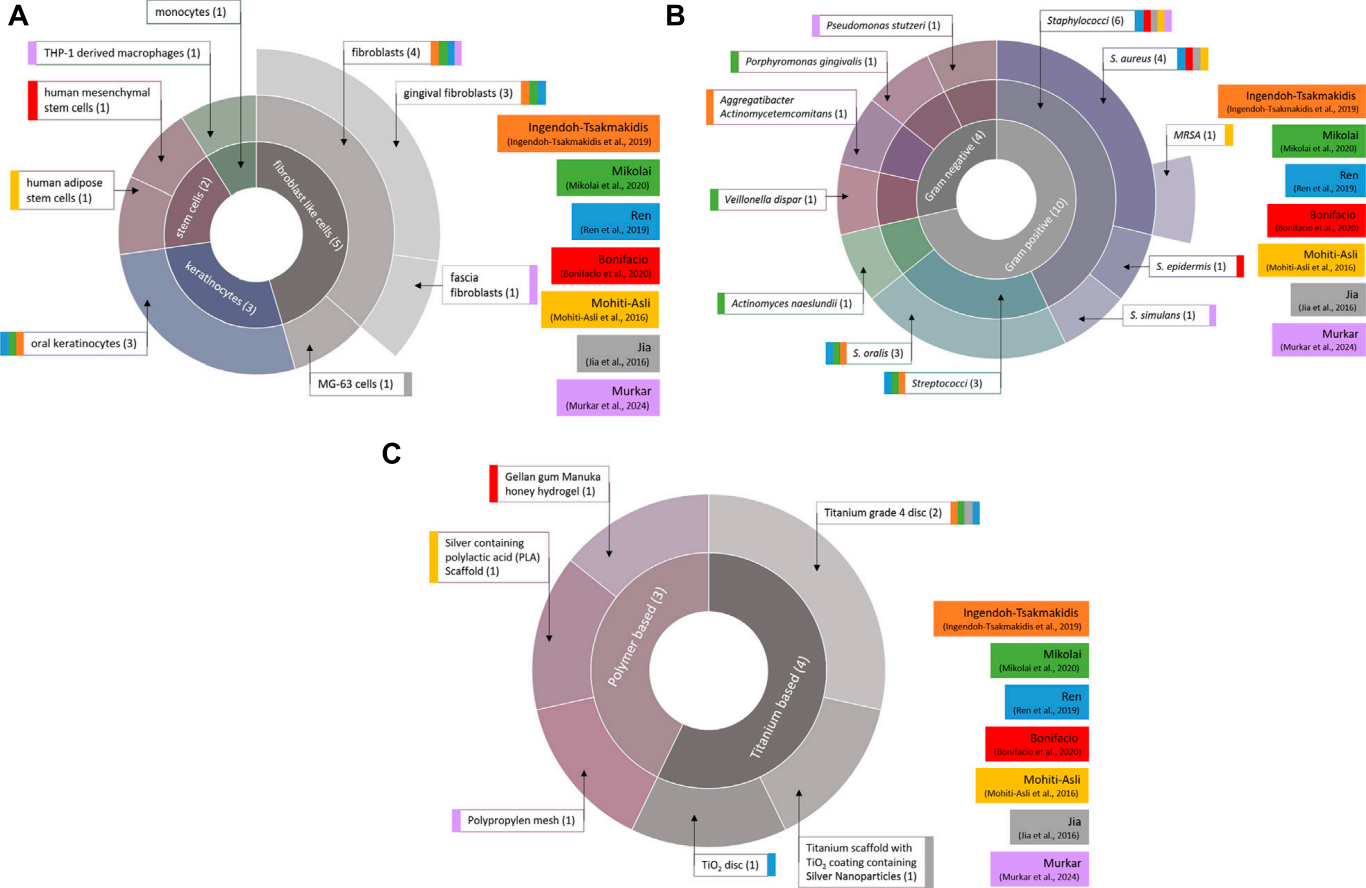
Distribution of cell types **(a)**, bacterial species **(b)** and implant materials **(c)** used in the 3D models. Total number of applications of the respective component are written in brackets. Colored blocks indicate the corresponding study.

Figure 2 shows an overview of the cell types (Figure 2a), bacterial species (Figure 2b) and implant materials (Figure 2c) used in all studies. In total, seven different cell types, twelve different bacterial species and six different implant materials were used in the described models.

Most of the authors built the 3D model on the basis of a 3D porous scaffold, but only Ren et al. used a transwell system where the integrated cells were separated by a semipermeable membrane (Ren et al., 2019). Depending on the tissue engineering (TE) approach, the scaffold was either the implant itself (implant-TE) or was intended to mimic the extracellular matrix of native tissues (ECM) for setting up organotypic models for *in vitro* testing (organotypic-TE). The scaffold for organotypic 3D models was either a bovine collagen type 1-based hydrogel (Ingendoh-Tsakmakidis et al., 2019; Mikolai et al., 2020) or a collagen-based scaffold derived from decellularized pig intestine [Small Intestinal Submucosa Segments (SIS-muc)] (Murkar et al., 2024). The remaining authors investigated 3D cell cultures on scaffold-structured materials, which were directly developed as implant materials (implant-TE). Those materials comprise a titanium scaffold coated with TiO_2_ containing silver nanoparticles (Jia et al., 2016), a silver ion-releasing polylactic acid (PLA) scaffold (Mohiti-Asli et al., 2016) and a gellan gum hydrogel with Manuka honey and inorganic clays (Bonifacio et al., 2020).

The focus of the included studies was only partially on model development. Most studies also performed experiments on the implant materials themselves as well as single cultures of cells and/or bacteria that were not integrated in this review.

#### 3.2.1 Dental implant models

The culture conditions of cells and bacteria of dental implant infection models are shown in Table 3. In all of the three studies, human gingival fibroblasts and human oral keratinocytes were used. For bacterial challenge, *S. oralis* was used in all studies. Ingendoh-Tsakmakidis et al. additionally tested *A. actinomycetencomitans* (Ingendoh-Tsakmakidis et al., 2019) and Ren et al. also investigated *S. aureus* (Ren et al., 2019). Mikolai et al. performed their experiments with a multispecies biofilm containing *S. oralis*, *A. naeslundii*, *V. dispar* and *P. gingivalis* (Mikolai et al., 2020). Ingendoh-Tsakmakidis et al. and Mikolai et al. used bacterial biofilms whereas Ren et al. applied planktonic bacteria. All studies used titanium as implant material, either in the form of inserted titanium grade 4 cylinders (Ingendoh-Tsakmakidis et al., 2019; Mikolai et al., 2020) or in the form of supporting TiO_2_ discs (Ren et al., 2019). Ren et al. investigated the sensitivity of their model to different materials by further applying hydroxyapatite and silicon rubber (Ren et al., 2019).

**TABLE 3:** Co-Culture conditions of 3D models for dental implant infection research. “suppl.” = supplemented with.

	Ingendoh-Tsakmakidis et al. (2019)	Mikolai et al. (2020)	Ren et al. (2019)
Keratinocyte culture medium	KerSFM medium suppl. with CaCl_2_, EGF, BPE and penicillin/streptomycin	KerSFM medium suppl. with CaCl_2_, EGF, BPE and penicillin/streptomycin	Oral Keratinocyte Medium suppl. with oral keratinocyte growth supplement
Fibroblast culture medium	DMEM suppl. with fetal bovine serum (FBS) and penicillin/streptomycin	DMEM suppl. with FBS and penicillin/streptomycin	DMEM suppl. with FBS and ascorbic acid-2-phosphate
3D culture technique	Collagen-based scaffold with fibroblasts and integrated titanium, keratinocytes seeded on top at day 8. Models raised to air-liquid interface 4 days after keratinocyte seeding and cultivated in Airlift medium for further 13 days	Collagen-based scaffold with fibroblasts and integrated titanium, keratinocytes seeded on top. Models raised to air-liquid interface 4 days after keratinocyte seeding and cultivated in Airlift medium for further 13 days	Transwell system with keratinocytes growing on a membrane filter, underneath fibroblasts growing on implant material
Bacteria species	*S. oralis* and *A. actinomycetencomitans* as monospecies biofilms	*S. oralis, A. naeuslundii, V. dispar* and *P. gingivalis* mixed at equal volumes to form a multispecies biofilm	*S. oralis* and *S. aureus* separate and mixed as planktonic cells
Bacterial challenge	Medium replaced by antibiotic-free Airlift medium. Adult biofilm of each species placed on spacers over the cell culture with direct contact to titanium Co-culture time 24 h	Medium replaced by antibiotic-free Airlift medium +10% BHI + Vitamin K. Adult biofilm of mixed species placed on spacers over the cell culture with direct contact to titanium Co-culture time 24–48 h	72 h after seeding the cells on substratum/membrane each bacterial strain was added separately in suspension to the medium of the transwell system Co-culture time 24 h
Sketch of the model	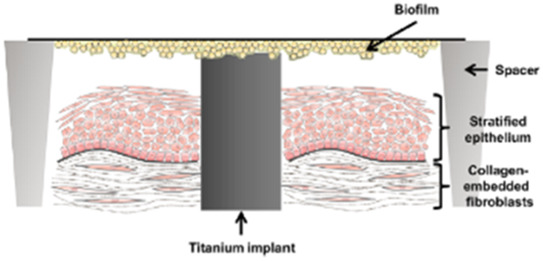 From Ingendoh-Tsakmakidis et al. (2019)	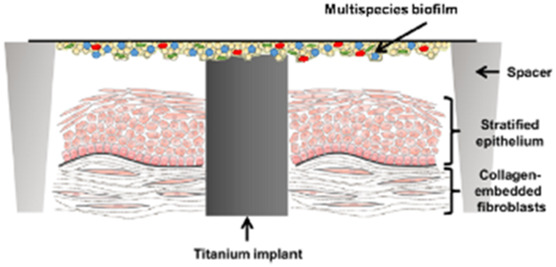 modified from Ingendoh-Tsakmakidis et al. (2019)	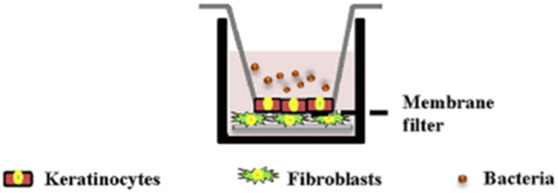 from Ren et al. (2019)

#### 3.2.2 Musculoskeletal implant models

The different setups for the three musculoskeletal implant models are displayed in Table 4. All authors used their experimental implant material as scaffolds for 3D cell cultures and challenged the cells with media containing staphylococcal bacteria. Mohiti-Asli et al. set up a 3D cell culture model to investigate their self-developed antibacterial scaffold for osteomyelitis treatment (Mohiti-Asli et al., 2016). Another model was established by Jia et al. to examine an antibacterial titanium scaffold to repair large scale bone defects. The 3D model of Bonifacio et al. (2020) is dedicated to investigate the biocompatibility and antibacterial properties of their scaffold for cartilage repair. The scaffold consists of gellan gum hydrogel supplemented with Manuka honey and different inorganic clays (mesoporous silica, halloysite nanotubes or sodium-calcium bentonite).

**TABLE 4:** Co-Culture conditions of 3D models for musculoskeletal implant infections.

	Mohiti-Asli et al. (2016)	Jia et al. (2016)	Bonifacio et al. (2020)
Scaffold material	PLA nanofibrous scaffold coated with Silverdur ET for silver ion release	Macroporous titanium scaffold with a micro/nanoporous coating of TiO_2_ containing silver nanoparticles	Gellan gum hydrogel containing medical grade Manuka honey and either mesoporous silica or halloysite nanotubes or sodium-calcium bentonite
Cells and culture medium	Human adipose stem cells cultivated in Complete Growth Medium (CGM) suppl. with FBS, L-glutamine and penicillin/streptomycin	MG-63 cells cultivated in α-Minimum Essential Medium (MEM) suppl. with FBS and penicillin/streptomycin	Human mesenchymal stem cells cultivated in DMEM suppl. with FBS and penicillin/streptomycin
Bacteria species	Methicillin-resistant *S. aureus* (MRSA) as planktonic culture	*S. aureus* as planktonic culture	*S. aureus* and *S. epidermidis* as mixed planktonic culture
Bacterial challenge	24 h after seeding cells on scaffold, medium was replaced by osteogenic differentiation medium containing MRSA. Co-culture time 2 weeks	12 h after seeding cells on scaffold, medium was replaced by antibiotic-free α-MEM and *S. aureus* was added in suspension Co-culture time 4 h	Cells were seeded on scaffolds in antibiotic-free DMEM. After 24 h medium was replaced by bacteria containing DMEM with FBS. Co-culture time 48 h
Sketch of the model	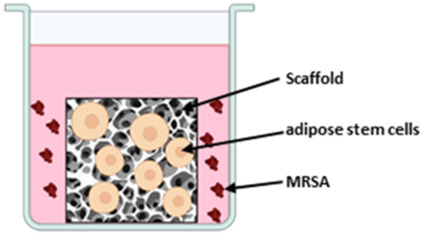 Created in BioRender. Winkel, A. (2025) https://BioRender.com/m38x480	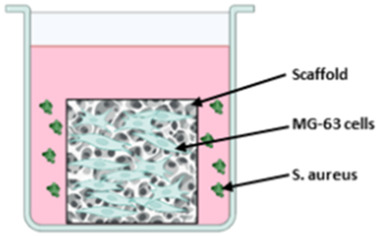 Created in BioRender. Winkel, A. (2025) https://BioRender.com/u56g716	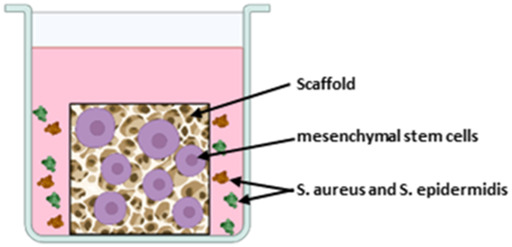 Created in BioRender. Winkel, A. (2025) https://BioRender.com/b58e007

#### 3.2.3 Immune cell-containing tissue model

The host’s immune response plays a pivotal role in clearing implant-associated infections. This includes innate immune cells such as macrophages and neutrophils, which phagocytose bacteria, secrete antibacterial proteins and reactive oxygen species and which also attract and stimulate other immune cells (Dong et al., 2022). This complex immune response is not yet reflected in *in vitro* models. A single model system has been introduced that includes macrophages and which thus can partially reflect immune responses *in vitro*. Macrophages are highly plastic cells that polarize to proinflammatory M1 states or anti-inflammatory and proreparative M2 states - depending on the environmental cues (Das et al., 2015). In their study, Murkar et al. (2024) established a 3D cell culture model containing fibroblasts and macrophages which were differentiated from the monocytic leukemia cell line THP-1. Table 5 provides an overview of the materials used and the methods for this model. The THP-1 derived M0 macrophages were subsequently polarized to M1 and M2 states. After seeding fibroblasts onto a SIS-muc scaffold and placing this into a medium containing macrophages (M0, M1 or M2), the cell cultures were challenged with an infected polypropylene mesh (Optilene^®^ Mesh Elastic, B. Braun, Melsungen, Germany).

**TABLE 5:** Co-culture conditions of an 3D immune cell-containing tissue model.

	Murkar et al. (2024)
Cells and culture medium	Fibroblasts from fascia biopsies cultivated in DMEM suppl. with FBS. THP-1 cells cultivated in THP-1 medium (RPMI-1640, L-glutamine, FBS, penicillin/streptomycin) and PMA. Differentiation into M0, M1 and M2 macrophages using specifically supplemented THP-1 medium
3D cell culture technique	Collagen based scaffold derived from porcine intestine (SIS-muc) seeded with fibroblasts on the apical side. After 24 h, scaffolds were placed within cell crown inserts into medium (50:50 THP-1 medium and fibroblast medium) with differentiated macrophages (either M0, M1 or M2) and cultured for 11 days
Bacteria species	*S. simulans* and *P. stutzeri* mixed and cultivated for 48 h on polypropylene mesh (implant material) for biofilm formation
Bacterial challenge	Bacteria loaded polypropylene meshes placed on top of the fibroblasts Co-culture time 3 days
Sketch of the model	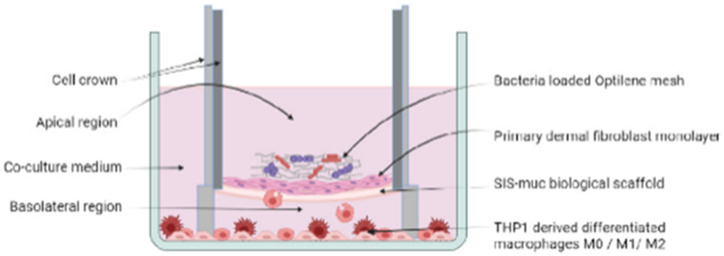 from Murkar et al. (2024)

### 3.3 Analytical methods after co-culture of cells and bacteria

All authors performed a variety of experiments to investigate specific characteristics of used materials, cells and bacteria before setting up their 3D cell-bacteria co-culture models. As the focus of this review is on 3D co-culture techniques, only those analytical methods that were used after co-culture of cells and bacteria in the presence of implant materials are described below.

**FIGURE 3:**
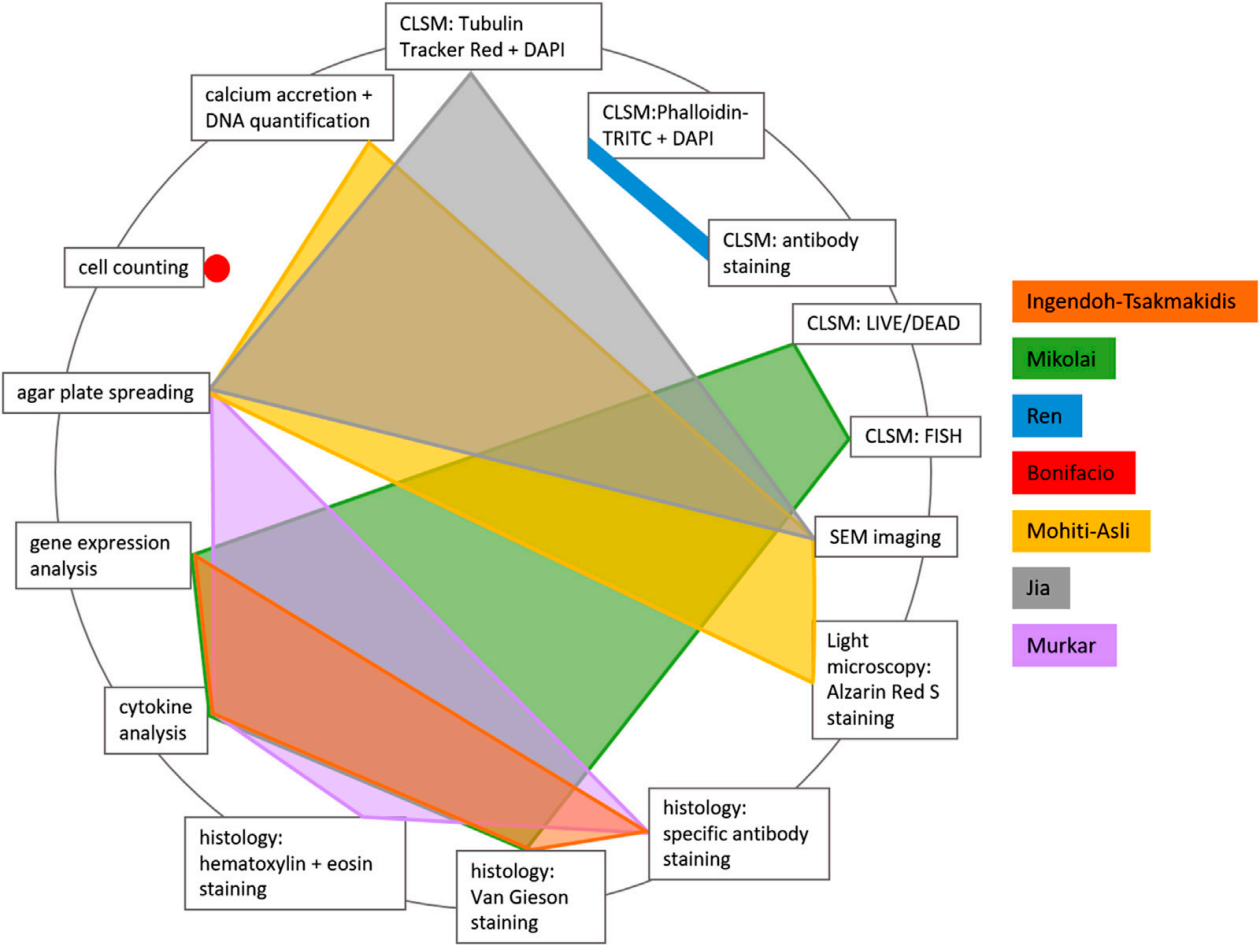
Distribution of analytical methods used after co-culture of cells and bacteria in 3D implant-associated models.

The methods used can be sorted into culture-based methods, functional assays, microscopic techniques, histology and molecular methods. The analytical methods used after co-culture of all 3D models are summarized in Table 6. Figure 3 shows the combinations of the individual analysis methods in each of the included publications.

**TABLE 6:** Analytical methods performed in each publication after co-culture of cells and bacteria.

Analytical methods		Staining	Study with applied method
Microscopy	CLSM imaging	Tubulin Tracker Red + DAPI	Jia et al. (2016)
		Phalloidin-TRICT + DAPI	Ren et al. (2019)
		Antibody staining	Ren et al. (2019)
		LIVE/DEAD	Mikolai et al. (2020)
		FISH	Mikolai et al. (2020)
	SEM imaging		Jia et al. (2016) and Mohiti-Asli et al. (2016)
	Light microscope imaging	Alzarin Red S	Mohiti-Asli et al. (2016)
Histology		Specific antibody	Ingendoh-Tsakmakidis et al. (2019) and Murkar et al. (2024)
		Van Gieson	Ingendoh-Tsakmakidis et al. (2019). and Mikolai et al. (2020)
		Hematoxylin + eosin	Murkar et al. (2024)
Molecular methods	Cytokine analysis		Ingendoh-Tsakmakidis et al. (2019), Mikolai et al. (2020), and Murkar et al. (2024)
	Gene expression analysis		Ingendoh-Tsakmakidis et al. (2019) and Mikolai et al. (2020)
Culture based methods	Agar plate spreading		Mohiti-Asli et al. (2016), Murkar et al. (2024), and Jia et al. (2016)
	Cell counting		Bonifacio et al. (2020)
Functional assays	Calcium accretion + DNA quantification		Mohiti-Asli et al. (2016)

Culture-based methods were used in four publications to quantify bacteria or cells. Bonifacio et al. used trypan blue staining of viable stem cells and a Burker chamber for cell counting (Bonifacio et al., 2020). Murkar et al. measured the turbidity of the surrounding medium in their 3D model and performed agar plate spreading of the medium from different culture conditions, in order to investigate differences in bacterial contamination (Murkar et al., 2024). Agar plate spreading and counting of colony forming units (CFU) was also performed by Mohiti-Asli et al., who harvested supernatant medium on each day of their 2 weeks of co-culture, in order to follow the development of bacterial contamination (Mohiti-Asli et al., 2016). Jia et al. used CFU counting on agar plates to quantify intracellular bacteria after cell detachment and lysis (Jia et al., 2016).

Only Mohiti-Asli et al. (2016) used a functional assay for measuring osteogenic differentiation of their 3D cultured stem cells. Total calcium secretion was measured with Calcium LiquiColor Kit and normalized to the total cell DNA, which was quantified with the DNA Hoechst fluorescence assay.

Three different microscopy techniques (light microscopy, scanning electron microscopy (SEM) and confocal laser scanning microscopy (CLSM)) were used in four included studies (Jia et al., 2016; Mohiti-Asli et al., 2016; Ren et al., 2019; Mikolai et al., 2020). Light microscopy was used by Mohiti-Asli et al. after staining their samples with Alzarin Red S for calcium visualization (Mohiti-Asli et al., 2016). Mohiti-Asli et al. (2016) and Jia et al. (2016) additionally investigated their osseous implant models with SEM imaging. CLSM imaging took place in three studies after different, partially multiple, staining procedures of either bacteria or cells. Mikolai et al. used LIVE/DEAD staining to calculate the total biofilm volume and to quantify viable and dead cells, together with FISH staining for quantification of the volume proportions of the species within the multispecies biofilm (Mikolai et al., 2020). Jia et al. quantified intracellular bacteria using FITC-labeled *S. aureus* for infection and staining the samples after cell lysis with Tubulin-Tracker Red and DAPI (Jia et al., 2016). Ren et al. stained cells with phalloidin-TRITC + DAPI and evaluated cell number and cell coverage of their transwell membrane and implant material in CLSM images. Additionally, antibody staining was used to visualize and quantify focal adhesions (Ren et al., 2019).

Histological analyses were performed by three authors (Ingendoh-Tsakmakidis et al., 2019; Mikolai et al., 2020; Murkar et al., 2024). After embedding the samples, they were cut or ground (when titanium was part of the model) into 5 µm (Ingendoh-Tsakmakidis et al., 2019; Mikolai et al., 2020) or 10 µm (Murkar et al., 2024) slices. Afterwards different staining techniques were applied. Mikolai et al. (2020) used van Gieson staining, as did Ingendoh-Tsakmakidis et al. (2019). The latter authors additionally performed specific antibody staining to visualize adherent junctions and the pro-inflammatory chemo- and cytokines IL-6, CXCL8 and TNF-α. Murkar et al. performed specific antibody staining for different cell types, combined with DAPI staining, and additionally used hematoxylin and eosin staining (Murkar et al., 2024).

Molecular methods included gene expression analysis performed by Ingendoh-Tsakmakidis et al. (2019) and Mikolai et al. (2020). They used RNA-based microarray analyses to identify changes in the cellular transcription profile in response to the bacterial challenge. Additionally, Ingendoh-Tsakmakidis et al. (2019), Mikolai et al. (2020), and Murkar et al. (2024) tested for cytokine secretion based on a Multiplex-Assay or Enzyme-linked Immunosorbent Assays (ELISA).

### 3.4 Open research knowledge graph

The dataset of this review is additionally published in the ORKG as an ORKG comparison (Brümmer et al., 2025). The ORKG comparison provides a structured and interactive overview of the data to compare the current 3D *in vitro* models for implant-associated infections, which include and analyze the interaction of cells in a 3D culture, infection-relevant bacterial strains, and implant material. This overview helps other researchers quickly understand the merits of different approaches. While the published ORKG comparison is stable and accessible over the long term through its DOI, ORKG comparisons are versionable so that they can be continuously (re-)used, updated, and expanded (Karras et al., 2024). When a new related publication on 3D *in vitro* models for implant-associated infections is published, the ORKG comparison can be easily update. The new publication only needs to be described regarding the extracted data in the ORKG, and added to the ORKG comparison. The updated ORKG comparison can be published as a new version. In this way, the data is openly accessible to other researchers in the long term to promote open science, replication, and reuse. For example, the ORKG comparison and its underlying data can be helpful to answer competency questions about the models and can also be used for supplemental visualizations. In the supplementary materials, three exemplary executable competency questions with corresponding answers and visualizations are provided to illustrate the data reuse.

### 3.5 Risk of bias assessment

**FIGURE 4:**
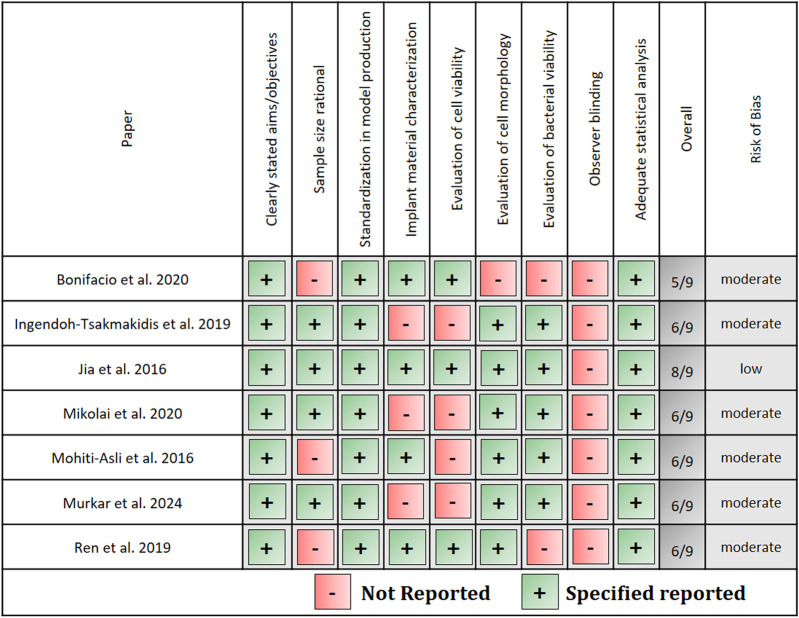
Risk of bias assessment.

Figure 4 presents the results of the risk of bias assessment. One study showed a low risk of bias and six studies showed a moderate risk of bias. No article provided information about observer blinding while no study showed a risk of bias concerning “stated aims” and “standardization of model production”. A sample size of N = 3, applied in four studies, was not evaluated rationally for assessment of biological questions (Mohiti-Asli et al., 2016; Ren et al., 2019). For establishment of a new model, a sample size of N = 3 was evaluated as the bare minimum (Jia et al., 2016; Murkar et al., 2024). Only two studies used greater sample sizes (Ingendoh-Tsakmakidis et al., 2019; Mikolai et al., 2020) and one study did not provide any information about the sample sizes (Bonifacio et al., 2020). Wherever the implant material was fabricated by the authors, it was characterized extensively. Of the authors following the organotypic TE approach, only Ren et al. characterized the used implant material in detail (Ren et al., 2019). Cell viability after co-culture with bacteria in presence of the implant material was only directly tested by two authors (Jia et al., 2016; Bonifacio et al., 2020) and assessed by Ren et al. through transepithelial electrical resistance measurement (Ren et al., 2019). Cell morphology was visualized by six authors and bacterial viability after co-culture was assessed in five studies.

## 4 Discussion

The application of complex 3D *in vitro* models to investigate implant-associated infections enlarges the understanding of host-microbe-implant interactions and allows us to investigate new therapy options and potential implant materials while reducing the number of *in vivo* experiments. This review aimed to provide a comprehensive overview on currently existing 3D *in vitro* models dedicated to implant-associated infections, including the three main compartments - tissue cells, implant material and infection-relevant bacteria. The systematic literature search revealed seven studies that met the inclusion criteria, published between 2016 and 2024. These relatively recent publication dates prove that 3D cell culture techniques have only been within the scientific focus since the early 2000s (Abbott, 2003) and are thus relatively new compared to classical cell culture techniques. 3D *in vitro* models are already being increasingly used to answer biological questions that do not concern implants. Nonetheless, the low number of relevant publications indicates the need for further development in the field of biomaterial-associated infections.

The basis for 3D *in vitro* models are soft or hard scaffolds that provide mechanical and biological properties close to the native tissue, in order to allow cell attachment, migration and differentiation (Andrée et al., 2013). Depending on the TE approach, the scaffold is the implant itself (implant-TE) or is intended to mimic the native tissues’ extracellular matrix (ECM) when setting up organotypic models for *in vitro* testing (organotypic- TE). Both approaches can be found within the included publications: three studies followed implant-TE approaches by developing new implant materials for different tissues (Jia et al., 2016; Mohiti-Asli et al., 2016; Bonifacio et al., 2020). The other four studies performed organotypic-TE by developing 3D organotypic models applicable to different implant materials to analyze cell behavior with and without bacterial challenge (Ingendoh-Tsakmakidis et al., 2019; Ren et al., 2019; Mikolai et al., 2020; Murkar et al., 2024). For the latter purpose, cells were cultivated on scaffolds mimicking the ECM (Ingendoh-Tsakmakidis et al., 2019; Mikolai et al., 2020; Murkar et al., 2024) or in a transwell system (Ren et al., 2019) and the implant material was incorporated into the model. Ingendoh-Tsakmakidis et al. and Mikolai et al. used a collagen-type 1 based hydrogel into which fibroblasts were already mixed in a liquid state (Ingendoh-Tsakmakidis et al., 2019; Mikolai et al., 2020). Collagen is a fibrous protein, a major component of animals’ and humans’ ECM in most tissues, such as dermis, gingiva and bone (Rezvani Ghomi et al., 2021). Thus, the application of collagen in biomaterial science is common, and it is widely used as scaffold material in 3D cell culture models (Redmond et al., 2021; Rezvani Ghomi et al., 2021). However, attention must be paid to the structure of the collagen-based matrix, which can differ significantly between different tissues. To ensure fibroblast growth within the collagen mixture, the authors added L-glutamine, FBS and DMEM for nutrition. Murkar et al. seeded their fibroblasts on Small Intestinal Submucosa Segments (SIS-muc scaffolds), which are decellularized parts of pork jejunum containing cross-linked collagen I, III and VI (Murkar et al., 2024). Depending on the decellularization and sterilization protocol, SIS-muc might also contain bioactive factors, e.g., cell adhesion factors, mitogenic factors and chemotactic cytokines. In comparison to native tissue, this gives the material an advantage over alloplastic collagen (Yang et al., 2010). However, the thickness of the SIS-muc and, thus, of the engineered tissue is limited by the anatomy of the jejunum. It usually measures around 100–200 µm and thus is approx. 10-fold thinner than artificial collagen hydrogels (Mertsching et al., 2005; Andrée et al., 2013). Moreover, the composition and mechanical properties of pork jejunum do not correspond with most human tissues. Therefore, the scaffold composition and properties should always be considered when selecting an appropriate 3D *in vitro* model for a specific scientific question.

In contrast to scaffold-based 3D cell cultivation, Ren et al. grew keratinocytes on a polyethylene terephtalate membrane filter in a transwell system (Ren et al., 2019). Underneath the porous membrane, fibroblasts were cultivated on top of the implant material. Such transwell setups make it possible for different cell types to grow in a tissue-like layered way within close distances to each other. In the study of Ren et al., the membrane could be considered an imitation of the basal membrane that separates keratinocytes and fibroblasts in the natural gingiva. Pores in the transwell membrane enable secondary metabolites to cross the membrane and ensure communication between the cells. This is also important because in the case of *in vivo* inflammation, cell-cell junctions between keratinocytes loosen, and thus allow immune cells and mediators to pass towards the epithelium. The increased permeability also enables bacteria to penetrate the epithelial cell layers on the other hand (Groeger and Meyle, 2015). Ren et al. tested different pore sizes (0.4 μm and 3 µm) during the setup of their model and concluded that 3 µm pores are needed for physiological cell-cell contact between the two layers as well as for bacterial passage, which is in line with the literature (Tyrer et al., 2011; Klein et al., 2013; Kusek et al., 2014). However, in contrast to scaffolds, the transwell system does not provide a structure for cells to grow in a 3D morphology. Such models can therefore be considered the most straightforward form of 3D *in vitro* models.

In the human body, every process is embedded in a fluidic dynamic that static *in vitro* models are lacking. Recently, in various fields, these microfluidic influences are being incorporated into 2D and 3D cell culture models with the benefit of miming the realistic conditions even better. Those advantages lay the ground for the development of complex models, meaning organoids on a chip (Saorin et al., 2023). None of the included studies followed a microfluidic approach in their experimental setup. The incorporation of fluidic dynamics into the already complex 3D models for implant associated infections would be a great advantage in the future.

### 4.1 Dental implant models

Three of the included studies described dental implant 3D *in vitro* models. Dental implants are osseointegrated in the alveolar bone and pass through the connective and epithelial tissue into the oral cavity, where the superstructure is fixed onto the implant to replace missing teeth. Gingival keratinocytes and fibroblasts are connected to the implant surface via a basal membrane with cell junctions such as hemidesmosomes and focal adhesions (Fischer and Aparicio, 2022).

The 3D models for investigation of dental implant infections all included keratinocytes and fibroblasts as the most important cells of the oral soft tissue. Ren et al. grew fibroblasts on top of the dental implant material below the transwell membrane that holds the keratinocyte layer. Bacteria where added to the medium, surrounding the keratinocytes (Ren et al., 2019). The authors conclude that within their model, keratinocytes protect fibroblasts from bacteria as in the natural tissue. However, the setup lacks a direct connection between keratinocytes and the implant material, which is the major location for bacterial invasion. Among other factors, the integrity of the keratinocyte-implant-junction depends on the implant material properties and is usually weaker than the cell-cell junctions (Jin et al., 2017; Fischer and Aparicio, 2022). Consequently, if inflammation develops, the epithelial attachment towards the implant is the first to be loosened and bacteria can invade in the apical direction. The influence of material properties on this process cannot be analyzed in the described transwell model. However, this model is well suited to analyze the cross-talk of the 2 cell types upon bacterial challenge in the presence of implant materials. In contrast, within the models of Ingendoh-Tsakmakidis et al. and Mikolai et al., keratinocytes and fibroblasts both have direct contact to the implant surface and to each other (Ingendoh-Tsakmakidis et al., 2019; Mikolai et al., 2020). Furthermore, keratinocytes were grown in realistic conditions at an air-liquid interface which allows them to differentiate and form a multilayered epithelium. This allows direct analysis of how implant properties influence epithelial detachment, but limits the separate analysis of the 2 cell types. Additionally, both model systems focus on the peri-implant soft-tissue. The implant-supporting bone, vascularization or immune cells have not been considered so far, even though they play an important role in peri-implant infections. Integration of these structures in future would greatly increase the informative value of dental implant 3D *in vitro* models and strengthen their benefit for novel therapeutic and preventive approaches.

With regard to tissue-bacteria interaction, Ingendoh-Tsakmakidis et al. found that the destructive effect on the tissue differs between bacterial species. This highlights the importance of choosing appropriate bacterial species for investigation of peri-implant infections. In all dental models, the Gram positive *S. oralis*, a major commensal pathogenic bacterium of the oral cavity, was used. Furthermore, Ren et al. applied *S. aureus* to their model. Carvalho et al. state in their meta-analysis from 2023 that *S. aureus* is not associated with peri-implantitis but, nevertheless, is a bacterium frequently found in the oral cavity that impairs oral keratinocytes and fibroblasts (Ren et al., 2019; Carvalho É et al., 2023). The meta-analysis also indicates that the Gram negative bacterium *A. actinomycetencomitans* is not associated with peri-implantitis. This is consistent with the finding of Ingendoh-Tsakmakidis et al., who could not find cell destruction in the presence of *A. actinomycetencomitans* biofilms. However during the formation of periodontitis *A. actinomycetencomitans* favors the colonization with pathogenic species (Groeger and Meyle, 2019). This shows as an example that bacterial strains must also be selected taking into account the interactions between different bacteria in a multispecies biofilm, as occurs in patients. Mikolai et al. placed a multispecies biofilm, containing different oral bacteria, on top of the implant. According to its species distribution, this multispecies biofilm is associated with oral health. The setup with a multispecies biofilm better mimics the *in vivo* situation, both in terms of the variety of microorganisms present and the higher virulence of biofilms compared to planktonic bacteria (Kommerein et al., 2017). Nevertheless, depending on the research question the application of a pathogenic biofilm associated with peri-implantitis would be desirable. *In vivo* infections of dental implants are complex, involve many different cell types and bacterial species and additionally depend on the microfluidic environment. As the reproduction of this complexity *in vitro* is still limited in the described models, 3D models for peri-implantitis benefit greatly from using relevant bacterial strains, preferably also in a multispecies mixture and taking biofilm tolerance into account. In the future, 3D peri-implantitis models could additionally consider integration of pathogenic biofilms or even more complex individual patient samples.

### 4.2 Musculoskeletal implant models

All three studies that developed the 3D musculoskeletal implant infection models followed implant-TE approaches for development of antibacterial implantable scaffolds (Jia et al., 2016; Mohiti-Asli et al., 2016; Bonifacio et al., 2020). For 3D cell culture, the scaffolds were seeded either with human adipose mesenchymal stem cells (Mohiti-Asli et al., 2016), bone marrow derived mesenchymal stem cells (Bonifacio et al., 2020) or with MG-63 cells, osteoblast-like human osteosarcoma cells (Jia et al., 2016). The tumor-derived cell line MG-63 is widely used as an osteoblast-like cell in biomaterial research because of its good availability and its potential for producing bone-associated proteins. However, these cells differ from primary osteoblasts in matrix composition (Pautke et al., 2004) as well as in the production of cytokines, chemokines and growth factors (Mussano et al., 2017). In contrast, stem cells are closer to the *in vivo* situation, but are more difficult to obtain and cultivate as they need to be freshly isolated and can only be used for certain passages. However, since adipose tissue-derived stem cells can be used as source of mesenchymal stem cells, their application for 3D *in vitro* models is facilitated.

Co-culture of cell containing scaffolds and bacteria was performed using *S. aureus* in all models, with Mohiti-Asli et al. using a methicillin-resistant *S. aureus* (Jia et al., 2016; Mohiti-Asli et al., 2016; Bonifacio et al., 2020). *S. aureus* is the most commonly involved pathogen in orthopedic infections (Kapadia et al., 2016; Zeller et al., 2018) and its methicillin-resistant mutation has a high prevalence, making infection treatment even more difficult. Bonifacio et al. additionally mixed *S. aureus* with *S. epidermidis*, another common pathogens in prosthetic joint infections (Zeller et al., 2018). Therefore, the selected bacterial strains are highly relevant for orthopedic infections. Nevertheless all applied bacterial species are Gram positive but the prevalence of prosthetic joint infections caused by Gram negative bacteria ranges between 10% and 25% (Gonzalez et al., 2025). In future, probably Gram negative bacterial species such as *E. coli* or *pseudomonas* species and the Gram positive enterococci could additionally be added into the system as they also can be relevant in infections of orthopedic implants (Zeller et al., 2018). All authors added the bacteria in suspension to the medium surrounding the scaffold, either 12 h (Jia et al., 2016) or 24 h (Mohiti-Asli et al., 2016; Bonifacio et al., 2020) after cell seeding. Microorganisms can infect the musculoskeletal system through hematogenous spread or direct inoculation (Zeller et al., 2018). In both cases, the microorganisms can form biofilms on implant materials, but during infection they appear as planktonic bacteria. This infection route distinguishes them from dental implants and supports the use of planktonic bacteria in experiments for assessment of the antibacterial properties of the tested musculoskeletal implant models. Mohiti-Asli et al. performed a cell-bacteria co-culture for 14 days, which is a noticeably long time period compared to the other authors, who maintained co-culture for 4–48 h. The results can therefore provide information about established bacterial infections. However, when evaluating the cell/bacteria interaction in the models, investigating possible biofilm formation could provide additional insights.

Even though the used single cell type and two bacterial species are key components in prosthetic joint infections, the resulting 3D models are still a simplification of the clinical situation. Neither the influence of the immune system nor microfluidic effects can be investigated using the described models. The addition of further cell types and additional implant materials to the *in vitro* models would also require certain changes to the experimental setup. The developed systems are therefore more dedicated to investigate cell- and bacterial behavior in the presence of a specific material, and less towards understanding infection processes and interactions between biomaterials, cells and bacteria. An organotypic model for the investigation of implant-associated infections of bone and cartilage is currently still missing.

### 4.3 Immune cell-containing tissue model

The only immune cell-containing tissue model of this study was introduced by Murkar et al. to investigate cell behavior in biofilm presence (Murkar et al., 2024). The authors seeded fibroblasts on a SIS-muc scaffold that was fixed in cell crown inserts. Fibroblasts produce the ECM of connective tissue and are therefore important cells for mechanical tissue properties as well as tissue repair. Moreover, they maintain close contact to other tissue cells by mediator secretion during homeostasis, injuries or infection (Plikus et al., 2021). These features make fibroblasts a good representative of most tissues. However, the transferability of the described model to transdermal or transmucosal implants is limited due to the missing epithelial tissue that protects fibroblasts *in vivo* as described in 4.1. Underneath the scaffold material, the authors cultivated THP-1 derived macrophages. THP-1-derived macrophages showed similar behavior to human isolated macrophages in infection-related studies. Therefore, and because of their superior genetic homogeneity and longevity, in their systematic review of 2022, Yasin et al. recommend using THP-1 derived macrophages rather than primary human monocyte-derived macrophages (Yasin et al., 2022). However, given the recent progress in generating large numbers of primary macrophages from induced pluripotent stem cells (Ackermann et al., 2022), it can be expected that primary macrophages will move into focus. Macrophages are an important part of the innate immune response with the secretion of pro-inflammatory cytokines during bacterial infection. In addition, they fulfill a number of functions in tissue homeostasis (Wynn et al., 2013). With respect to implant-associated infections and bacteria defense, macrophages play an important role in tissue healing after implantation (Zhang et al., 2021) and their share in general bone remodeling has also been suggested (Wynn et al., 2013; Giraldo-Osorno et al., 2024). When establishing scaffold-based models, the influence of the scaffold materials themselves on immune cells, e.g., macrophages, must be considered (Sadtler et al., 2019).

Murkar et al. used an infected polyethylene mesh that was placed on top of the fibroblasts for bacterial challenge of the cells. In this way, the authors incorporated a representation of soft tissue wound-healing implants within the model. However, the application of this model to other implants is difficult due to the sensitivity of the cell crown construction to applied weight. The weight of, e.g., metallic implants, might jeopardize the scaffold’s integrity or exert unphysiological stimuli upon the cells. If other implant materials were used, the model of Murkar et al. would probably have to be redesigned. However, no other 3D model investigates the interaction of macrophages and tissue cells in the presence of an infected implant and this could be an important issue. If an organotypic 3D model could be developed that was transferable to different implant materials, this could support studies on the role of macrophages and even other immune cells.

### 4.4 Advantages and disadvantages of model setups

Table 7 displays the advantages and disadvantages of the different 3D *in vitro* model setups described in the included studies.

**TABLE 7:** Advantages and disadvantages of model setups.

3D Model setup	1st author	Advantages	Disadvantages
Organotypic dental implant model: fibroblasts containing scaffold with keratinocytes growing on top and integrated titanium in Airlift medium. Biofilm placed on top of the titanium implant	Ingendoh-Tsakmakidis, Mikolai	Keratinocytes form a multilayerds epithelium Interaction of fibroblasts and keratinocytes toward implant material possible Bacterial invasion along the implant close to clinical situation Biofilms show higher virulence then planktonic bacteria Multispecies biofilms also take interactions between different bacterial species into account (Mikolai et al., 2020)	Lack of bone tissue, immune cells and microfluidic dynamics Highly time consuming model setup Biofilm composition simplified compared to clinical situation Multispecies biofilm (Mikolai et al., 2020) reflects healthy state → pathogen biofilm missing
Transwell system with fibroblasts growing on implant material underneath a membrane filter and keratinocytes growing on top of the membrane	Ren	Cross-talk between cells without direct contact in presence of implant material can be studied High throughput and easy adaption to different implant materials possible Planktonic bacteria usage enables to change and mix bacterial species easily	No connection between keratinocytes and implant material → bacterial invasion not modeled realistically →impact of material properties on loosening of keratinocyte-implant interaction in case of inflammation cannot be studied Keratinocytes do not form multilayered epithelium Lack of bone cells, immune cells, microfluidic dynamic Planktonic bacteria are not reflecting clinical infection pathway of dental implants Higher virulence of biofilm compared to planktonic bacteria is not taken into account
Tissue engineered scaffold (developed to be implant material) seeded with cells and infected with bacteria containing medium	Bonifacio, Jia, Mohiti-Asli	Influence of material on cells and bacteria in Co-Culture can be evaluated High throughput during implant material development possible	No organotypic model to investigate infection routes ant interactions of cells, bacteria and materials Only 1 cell type used Lacking 3D tissue (e.g., bone tissue) into which the implant is incorporated Biofilm formation and higher virulence of biofilms not taken into account Implant materials not replaceable
Cell crown setup with THP-1 derived macrophages growing underneath a SIS-muc Scaffold with incorporated fibroblasts. Implant material is infected with biofilm and placed on top of SIS-muc	Murkar	Model includes immune cells Not specific for only one implant → outcomes applicable to different tissues Biofilm application takes higher virulence compared to planktonic bacteria into account Mixed-species biofilm takes interaction between bacterial species into account	No specified tissue addressed → further refinement for special tissues needed Makrophage reaction to scaffold material is not evaluated SIS-muc application limits height of the 3D model Model setup limits the application of implant materials with high weight

### 4.5 Analytical methods after co-culture of 3D cells and bacteria

For the purpose of this review, only those analytical methods were taken into account that were used after co-culture of cells and bacteria in the 3D models. Prior to co-culture, all authors validated cell numbers and distribution within their models. Most authors also performed assays to assess cell metabolism. Analytical methods of implant-infection 3D models should provide information about the viability, morphology and activity of cells after bacterial challenge, as well as the influence on cell-implant-interaction. Therefore, the combination of molecular methods (or functional assays) and microscopy, or histological imaging, seems suitable. The authors of the included studies used many different tests, ranging from only one test (Bonifacio et al., 2020) to five different tests (Mikolai et al., 2020). Most authors combined different methods to win an insight into cell- or bacterial numbers as well as activity and morphology. Mikolai et al. (2020), for example, quantified and visualized biofilm volume and composition using CLSM with two different staining methods. Cell activity during bacterial challenge was analyzed using gene expression analysis and cytokine analysis and tissue morphology was determined using histological sectioning, staining and imaging. In contrast, Bonifacio et al. (2020) only performed viable cell counting using trypan blue staining and a Burker chamber. Those straightforward methods for cell counting come with the advantage of low effort and costs but do not provide information about cell metabolism. Neither the bacterial influence on cell activity or morphologies could be taken into account, nor was bacterial growth investigated. When comparing implant-TE and organotypic-TE approaches, no difference in the number or type of analytical methods was observed. Even though the focus of implant-TE models often lies on investigating the developed materials themselves, most authors also used several cell-culture analyses. Mohiti-Asli et al. (2016), for instance, developed an *in vitro* 3D model and quantified bacteria as well as cells through DNA quantification. Furthermore, they quantified and visualized calcium deposition of cells, giving insight into relevant cell activity and visualized the 3D tissue through SEM imaging. Ren et al. (2019), in contrast, analyzed different parameters within one single method. By combining CLSM with different staining protocols, they won insights into general cell distribution and morphology as well as focal adhesion number and distribution. Cell metabolism after bacterial challenge or bacteria growth have not been investigated. The diversity of applied analytical methods indicates the diverse questions that were addressed by the reviewed models. In general, a combination of imaging methods and methods that assess cell metabolism, like molecular methods and functional assays, could be recommended and have mostly been used to draw conclusions on the bacterial influence on tissue-like grown cells. For insight into the tissue-implant interface of organotypic-TE 3D models, histological methods using appropriate staining procedures seem to be favorable.

## 5 Conclusion and outlook

Increasing implant usage and rising numbers of implant-associated infections lead to a need for suitable *in vitro* models in order to understand host-pathogen-implant relations and to test newly developed implant materials and diagnostic and treatment approaches. Therefore, currently available 3D tissue models that include relevant cells, bacteria and implant materials have been reviewed in this study. After a systematic literature search, only seven studies with six different models could be included. Those models constitute dental implant models, musculoskeletal implant models and a general immune cell-containing model. For skeletal implant infections, only implant-TE approaches were pursued, whereas for dental implants and for the immunocompetent model organotypic models were developed. All models fulfilled their purpose and allowed a specific readout of different implant-associated infection aspects. However, even though all 3D models depicted the natural situation more appropriately than classical 2D *in vitro* test systems do, this review highlights the need for further improvements of models for all implant fields. Particularly, organotypic infection models for skeletal implants are missing so far. In summary, to set up an organotypic model, a scaffold-based system should be used to create the desired 3D tissue, ideally including different tissue cell and immune cell types. To investigate the interaction of cells and implant materials, the arrangement of the implant with respect to the cells should as closely as possible mimic the *in vivo* situation. Due to the constantly improving methodology for preparing cells for *in vitro* experiments, it can be assumed that the complexity of the models will increase in the future. The bacterial species used in these models should be selected with respect to the implant studied, whereby for dental and musculoskeletal implants the application of biofilms instead of planktonic bacteria is more likely to reflect the clinical situation. For analytical methods, a variety of possibilities has been presented in the various studies. They should always be selected with regard to the scientific question. As a minimum, the analysis of cell numbers, cell activity and visualization of the implant-tissue interface is recommended to allow a statement about the influence of implant materials and bacterial load on tissue cells. Further progression and simplification of model production could even make it possible to personalize the 3D models with patient derived cells and/or biofilm samples in the future. Doing so, individual responses of patients to infections or materials could be studied. Therefore the further development of 3D *in vitro* models can contribute to a more personalized medicine in general and especially concerning implant-associated infections. To do justice to the rapid progression in the field of 3D *in vitro* models, the dataset of this review is published at the ORKG. This open science infrastructure makes it possible to supplement the ORKG comparison (Brümmer et al., 2025) of the prospectively published data on the scientific question of this review and ensures sustainable use of the data in the long term.

## Data Availability

The datasets for this review were additionally published at the Open Research Knowledge Graph (Brümmer et al., 2025) and can be accessed through this link: https://orkg.org/comparison/R1368153.
