# Supplementary material for: Better models, better treatment? a systematic review of current three dimensional (3D) in vitro models for implant-associated infections
Source: Front Bioeng Biotechnol. 2025 Apr 25;13:1569211. doi: 10.3389/fbioe.2025.1569211 (PMC12061920; doi:10.3389/fbioe.2025.1569211)
Supplement: Supplementary file 1 [file DataSheet2.pdf]

Supplementary Materials

1 ORKG Comparison

The published ORKG comparison “3D In Vitro Models for Implant-Associated Infections” (Brümmer et al., 2025) is a structured overview of the underlying extracted data from the analyzed publications of our literature review (Karras et al., 2024). It aims to provide an interactive overview of the data to compare the current 3D in vitro models for implant-associated infections, which include and analyze the interaction of cells in a 3D culture, infection-relevant bacterial strains, and implant material. This overview helps other researchers quickly understand the merits of different approaches. The data is openly accessible to other researchers in the long term to promote open science, replication, and reuse.

We also provide an exported version of the ORKG comparison in an additional file in the supplementary materials: “ORKG_comparsion_exported.pdf”.

2 Illustration of Data Reuse Using the ORKG SPARQL Endpoint

All data in the ORKG and thus the data of the published ORKG comparison “3D In Vitro Models for Implant-Associated Infections” (Brümmer et al., 2025) is openly accessible to anyone. We illustrate the data reuse using the ORKG SPARQL endpoint by asking and answering three competency questions.

1. *What co-culture times for the different 3D in vitro models are reported in the publications?*

This natural language question can be transformed into a SPARQL query (see Figure 1) that can be executed on the ORKG SPARQL endpoint (https://orkg.org/sparql).

```
PREFIX orkgr: <http://orkg.org/orkg/resource/>
PREFIX orkgc: <http://orkg.org/orkg/class/>
PREFIX orkgp: <http://orkg.org/orkg/predicate/>
PREFIX rdfs: <http://www.w3.org/2000/01/rdf-schema#>
PREFIX xsd: <http://www.w3.org/2001/XMLSchema#>
PREFIX rdf: <http://www.w3.org/1999/02/22-rdf-syntax-ns#>

#defaultView:BarChart
SELECT (STR(SAMPLE(?paper)) AS ?article) ?culture_time_hours ?paper WHERE {
  orkgr:R1366249 orkgp:compareContribution ?contrib.
  ?paper orkgp:P31 ?contrib.

  ?contrib orkgp:P166067 ?co_culture.
  ?co_culture orkgp:P166069 ?culture_time.
  ?culture_time orkgp:wikidata:P1181 ?numerical_value;
              orkgp:P110124 ?unit.
  BIND(xsd:integer(?numerical_value) as ?culture_time_hours)
}
```

Figure 1: SPARQL query for the first competency question.

We provide the following link to the directly executable query: https://tinyurl.com/ORKG-query1. Figure 2 shows the resulting view after opening the link. By clicking on the blue button with the white arrow, the query is executed to retrieve the data from the ORKG and visualized as a bar chart (see Figure 3).

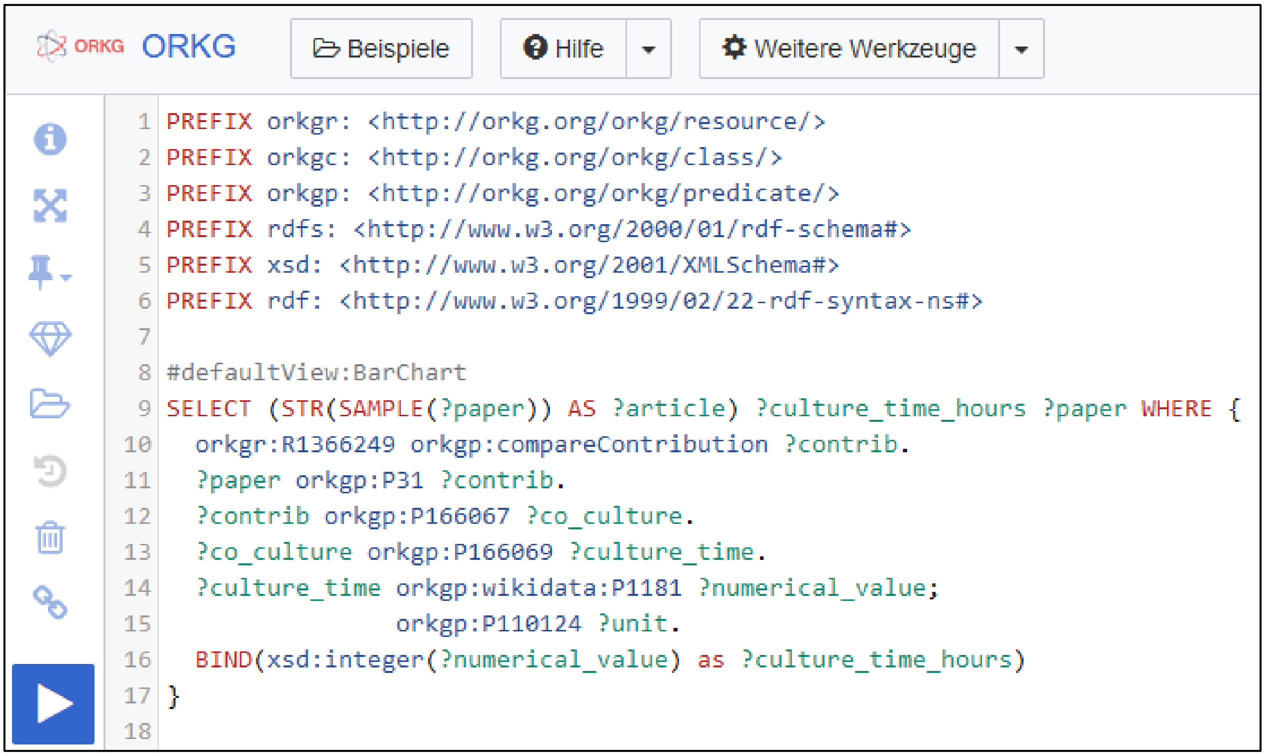

Figure 2: Executable ORKG query for the first competency question.

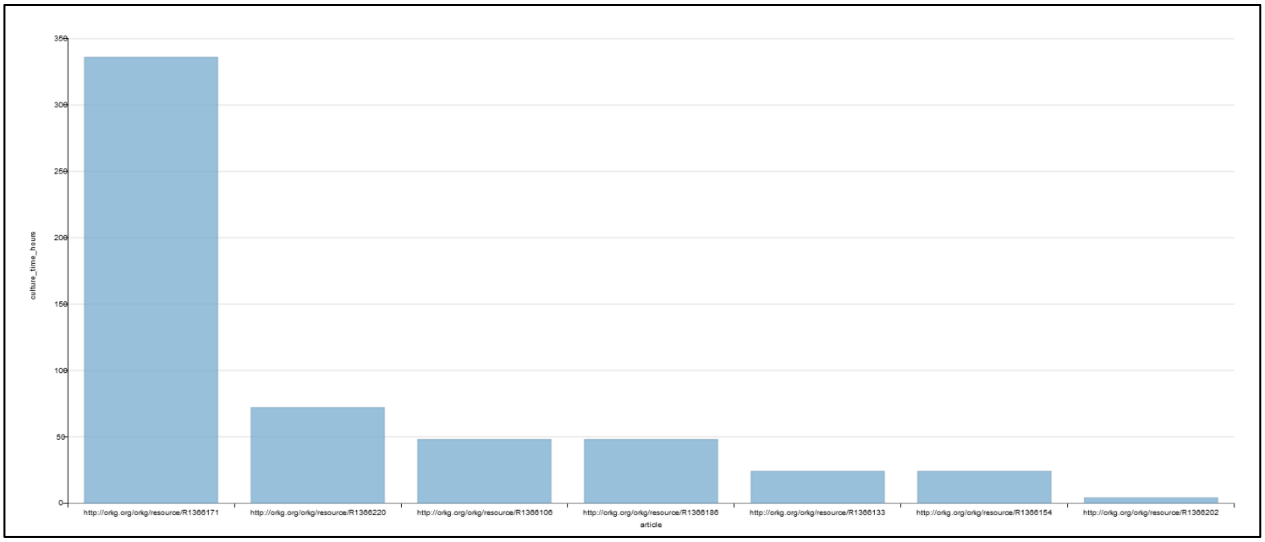

Figure 3: Resulting bar chart of executing the SPARQL query of the first competency question.

We can summarize this bar chart as follows to provide an answer to the first competency question:

Overall, the reported co-culture time ranges from 4 hours to 336 hours (two weeks), with an average of 79.43 hours, a median of 48 hours, and a standard deviation of 115.25 hours. While one publication reported a co-culture time of 4, 72, and 336 hours respectively, two publications reported a co-culture time of 24 and 48 hours respectively.

2. *Which analysis methods are used how often in the publications?*

This natural language question can be transformed into a SPARQL query (see Figure 4) that can be executed on the ORKG SPARQL endpoint (https://orkg.org/sparql).

```
PREFIX orkgr: <http://orkg.org/orkg/resource/>
PREFIX orkgc: <http://orkg.org/orkg/class/>
PREFIX orkgp: <http://orkg.org/orkg/predicate/>
PREFIX rdfs: <http://www.w3.org/2000/01/rdf-schema#>
PREFIX xsd: <http://www.w3.org/2001/XMLSchema#>
PREFIX rdf: <http://www.w3.org/1999/02/22-rdf-syntax-ns#>

#defaultView:BarChart
SELECT STR(SAMPLE(?method_name) AS ?method) COUNT(?method_name) ?method_name WHERE {
  orkgr:R1366249 orkgp:compareContribution ?contrib.
  ?paper orkgp:P31 ?contrib.
  ?contrib orkgp:P37450 ?analysis_resource.
  ?analysis_resource orkgp:P1005 ?analysis_method.
  ?analysis_method orkgp:SCHEMAORG:name ?method_name.
}
```

Figure 4: SPARQL query for the second competency question.

We provide the following link to the directly executable query: https://tinyurl.com/ORKG-query-2. Figure 5 shows the resulting view after opening the link. By clicking on the blue button with the white arrow, the query is executed to retrieve the data from the ORKG and visualized as a bar chart (see Figure 6).

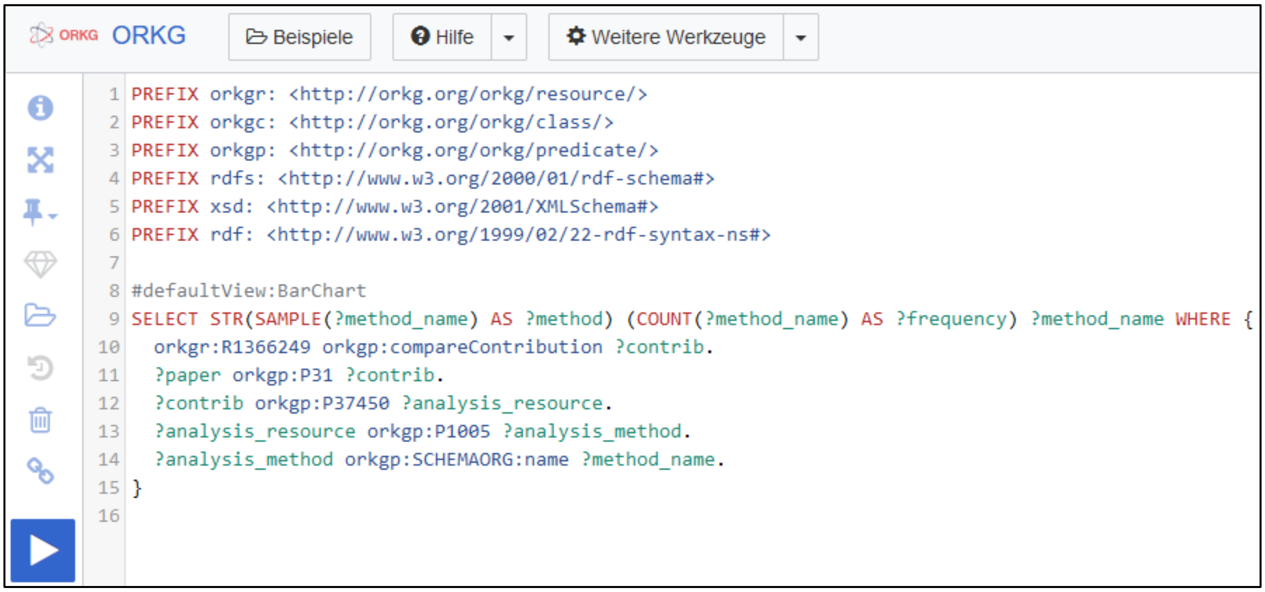

Figure 5: Executable ORKG query for the second competency question.

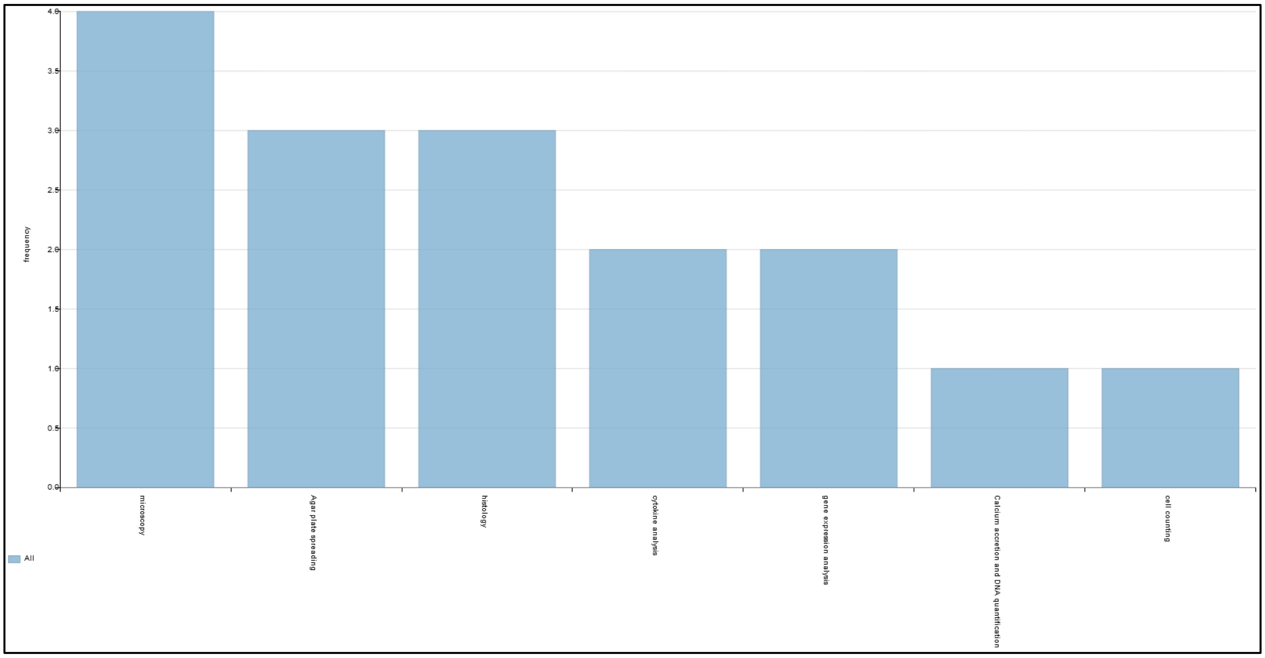

Figure 6: Resulting bar chart of executing the SPARQL query of the second competency question.

We can summarize this bar chart as follows to provide an answer to the second competency question:

In the publications, a total of 16 times seven different analysis methods are reported: 4x microscopy, 3x histology, 3x agar plate spreading, 2x cytokine analysis, 2x gene expression analysis, 1x calcium accretion and DNA quantification, 1x cell counting.

3. *How many analysis methods are reported per publication?*

This natural language question can be transformed into a SPARQL query (see Figure 7) that can be executed on the ORKG SPARQL endpoint (https://orkg.org/sparql).

```
PREFIX orkgr: <http://orkg.org/orkg/resource/>
PREFIX orkgc: <http://orkg.org/orkg/class/>
PREFIX orkgp: <http://orkg.org/orkg/predicate/>
PREFIX rdfs: <http://www.w3.org/2000/01/rdf-schema#>
PREFIX xsd: <http://www.w3.org/2001/XMLSchema#>
PREFIX rdf: <http://www.w3.org/1999/02/22-rdf-syntax-ns#>

#defaultView:BarChart
SELECT STR(SAMPLE(?paper) AS ?publications) (COUNT(?method_name) AS ?number_of_methods_per_paper)
?paper WHERE {
  orkgr:R1366249 orkgp:compareContribution ?contrib.
  ?paper orkgp:P31 ?contrib.
  ?contrib orkgp:P37450 ?analysis_resource.
  ?analysis_resource orkgp:P1005 ?analysis_method.
  ?analysis_method orkgp:SCHEMAORG:name ?method_name.
}
```

Figure 7: SPARQL query for the third competency question.

We provide the following link to the directly executable query: https://tinyurl.com/ORKG-query3. Figure 8 shows the resulting view after opening the link. By clicking on the blue button with the white arrow, the query is executed to retrieve the data from the ORKG and visualized as a bar chart (see Figure 6).

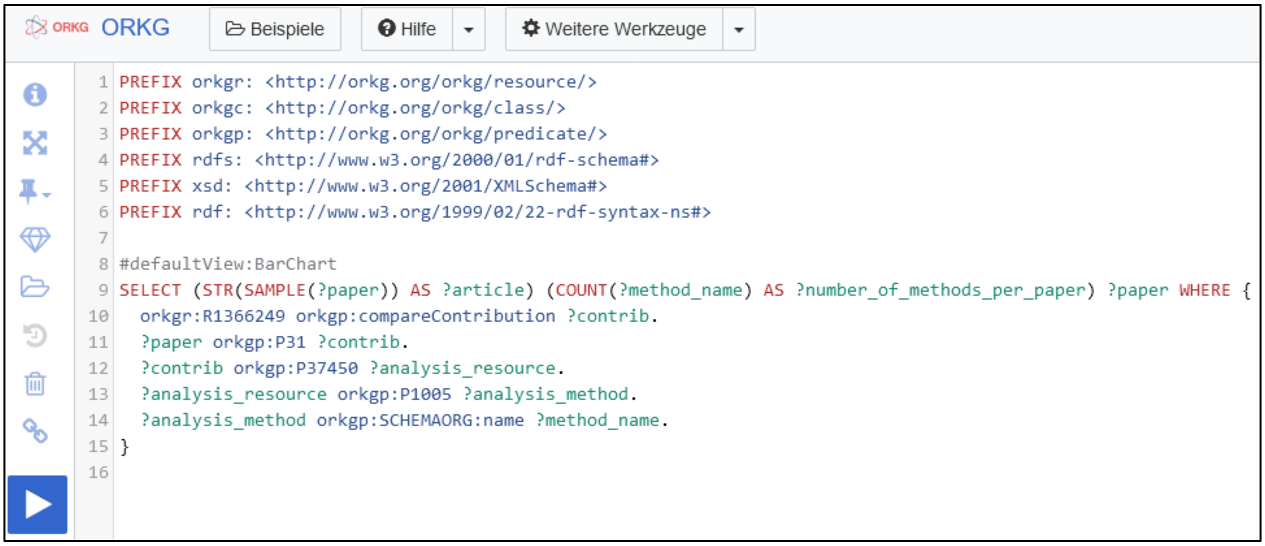

Figure 8: Executable ORKG query for the third competency question.

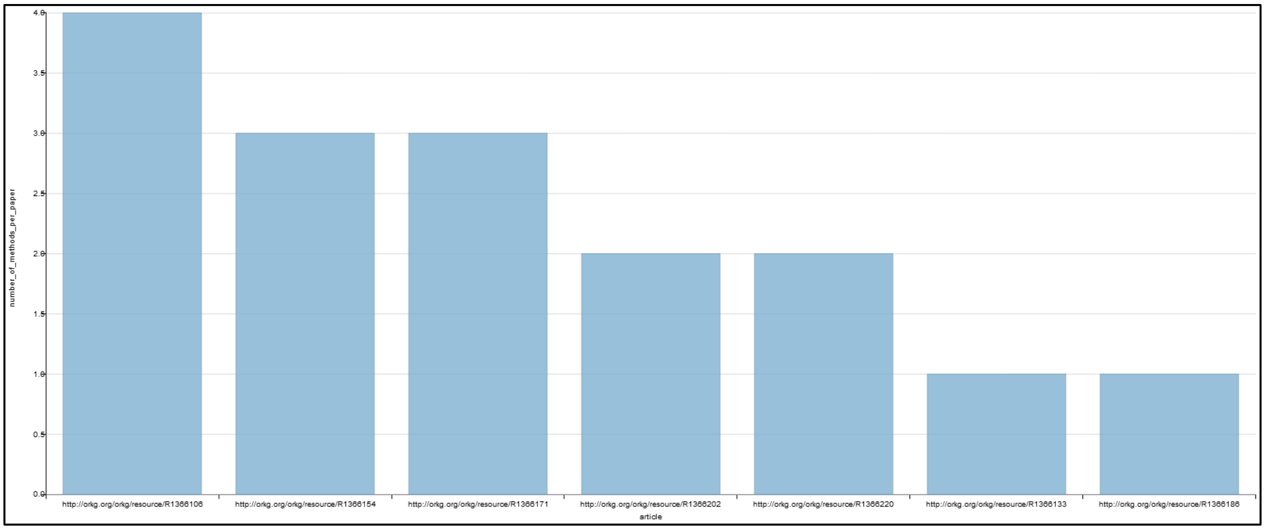

Figure 9: Resulting bar chart of executing the SPARQL query of the third competency question.

We can summarize this bar chart as follows to provide an answer to the third competency question:

Overall, the number of reported analysis methods per publication ranges from 1 to 4, with an average of 2.29, a median of 2, and a standard deviation of 1.11. While one publication reported 4 different analysis methods, two publications reported 3, 2, and 1 different analysis methods respectively.

These three examples show how the data from the published ORKG comparison (Brümmer et al., 2025) can be reused to get answers to competency questions related to the analyzed publications.

References

Brümmer, N., Doll-Nikutta, K., Schadzek, P., Mikolai, C., Kampmann, A., Wirth, D., et al. (2025). 3D In Vitro Models for Implant-Associated Infections. Open Research Knowledge Graph. doi: 10.48366/R1368153.

Karras, O., Budde, L., Merkel, P., Hermsdorf, J., Stonis, M., Overmeyer, L., et al. (2024). Organizing Scientific Knowledge from Engineering Sciences Using the Open Research Knowledge Graph: The Tailored Forming Process Chain Use Case. Data Science Journal 23(1). doi: 10.5334/dsj-2024-052.
