# Supplementary material for: Better models, better treatment? a systematic review of current three dimensional (3D) in vitro models for implant-associated infections
Source: Front Bioeng Biotechnol. 2025 Apr 25;13:1569211. doi: 10.3389/fbioe.2025.1569211 (PMC12061920; doi:10.3389/fbioe.2025.1569211)
Supplement: Supplementary file 2 [file DataSheet3.pdf]

Properties	Commensal and pathogenic biofilms differently modulate peri-implant oral mucosa in an organotypic model<br>*Contribution 1 - 2019*	Evaluation of Silver Ion-Releasing Scaffolds in a 3D Coculture System of MRSA and Human Adipose-Derived Stem Cells for Their Potential Use in Treatment or Prevention of Osteomyelitis<br>*Contribution 1 - 2016*	Keratinocytes protect soft-tissue integration of dental implant materials against bacterial challenges in a 3D-tissue infection model<br>*Contribution 1 - 2019*	Advances in cartilage repair: The influence of inorganic clays to improve mechanical and healing properties of antibacterial Gellan gum-Manuka honey hydrogels<br>*Contribution 1 - 2020*	Additively Manufactured Macroporous Titanium with Silver-Releasing Micro-/ Nanoporous Surface for Multipurpose Infection Control and Bone Repair – A Proof of Concept<br>*Contribution 1 - 2016*	Establishment of a Human Immunocompetent 3D Tissue Model to Enable the Long-Term Examination of Biofilm–Tissue Interactions<br>*Contribution 1 - 2024*	Early host–microbe interaction in a peri-implant oral mucosa-biofilm model<br>*Contribution 1 - 2020*
objective	Development of an oral mucosa model in 3D with an integrated implant challenged with oral bacteria and investigate interaction between bacteria and cells.	Development of a scaffold that sets free antibacterial silver for usage in osteomyelitis	Development of an 3D oral mucosa model with implant material integrated to investigate the soft seal around implant materials under bacterial challenges and with different materials used.	Development of an gellan-gum based hydrogel with manuka honey and different clays for replacement and repair of cartilage.	Development of a scaffold to repair large scale bone defects that is long term antibacterial and induces osseointegration.	Development of an immunocompetent 3D tissue model to investigate the influence of biofilms on tissue remodeling in human tissues.	Investigate interaction between oral mucosa and an early commensal multispecies biofilm using the 3D mucosa model.
implant model/implant model							
type*	dental implant	musculoskeletal implant	dental implant	musculoskeletal implant	musculoskeletal implant	unspecified	dental implant
category*	organotypic model	implant development	organotypic model	implant development	implant development	organotypic model	organotypic model
cell culture/cell culture							
3d scaffold*	Collagen type 1	PLA nanofibrous scaffold coated in polymer containing silver nitrate and releasing silver ions	terephthalate membrane filter in a transwell system	gellan gum with medical grade Manuka honey and mesoporous silica or holloysite nanotubes or sodium-calcium bentonite	macroporous titanium scaffold with a micro/nanoporous coating of TiO2 with silver nanoparticles	Fetal Bovine Serum (FBS)<br>Fetal Bovine Serum (FBS)<br>Fetal Bovine Serum (FBS)<br>Fetal Bovine Serum (FBS)<br>collagen-based matrix from pig intestine (SIS-muc)	Collagen type 1
cell type/cell type							
name*	yeast extract<br>yeast extract<br>yeast extract<br>yeast extract<br>human gingiva fibroblasts<br>human oral keratinocytes	human adipose stem cells	human gingiva fibroblasts<br>human oral keratinocytes	Luria Bertani broth<br>Luria Bertani broth<br>Luria Bertani broth<br>Luria Bertani broth<br>human mesenchymal stem cells	MG-63 cells	CASO Bouillon<br>CASO Bouillon<br>CASO Bouillon<br>CASO Bouillon<br>human primary fibroblasts<br>THP-1 cells	Brain heart infusion (BHI)<br>Brain heart infusion (BHI)<br>Brain heart infusion (BHI)<br>Vitamin K<br>Brain heart infusion (BHI)<br>Brain heart infusion (BHI)<br>Brain heart infusion (BHI)<br>Vitamin K<br>Brain heart infusion (BHI)<br>Brain heart infusion (BHI)<br>Brain heart infusion (BHI)<br>Vitamin K<br>Brain heart infusion (BHI)<br>Brain heart infusion (BHI)<br>Brain heart infusion (BHI)<br>Vitamin K<br>human gingiva fibroblasts<br>human oral keratinocytes
culture medium ingredient*	Bovine Pituitary Extract (BPE)<br>Calcium chloride<br>Dulbecco Modified Eagle's Medium (DMEM)<br>Epidermal growth factor (EGF)<br>Fetal Bovine Serum (FBS)<br>KerSFM medium<br>Show 2 more	complete growth medium<br>Fetal Bovine Serum (FBS)<br>L-glutamine<br>Penicillin/Streptomycin	Dulbecco Modified Eagle's Medium (DMEM)<br>Fetal Bovine Serum (FBS)<br>oral keratinocyte growth supplement<br>Oral Keratinocyte Medium (OKM)	Dulbecco Modified Eagle's Medium (DMEM)<br>Fetal Bovine Serum (FBS)<br>Penicillin/Streptomycin	Fetal Bovine Serum (FBS)<br>Minimum Essential Medium α (α-MEM)<br>Penicillin/Streptomycin	Fetal Bovine Serum (FBS)<br>Fetal Bovine Serum (FBS)<br>Fetal Bovine Serum (FBS)<br>Fetal Bovine Serum (FBS)<br>differentiation supplements<br>Dulbecco Modified Eagle's Medium (DMEM)<br>Fetal Bovine Serum (FBS)<br>Fetal Bovine Serum (FBS)<br>L-glutamine<br>Penicillin/Streptomycin<br>Show 2 more	Bovine Pituitary Extract (BPE)<br>Calcium chloride<br>Dulbecco Modified Eagle's Medium (DMEM)<br>Epidermal growth factor (EGF)<br>Fetal Bovine Serum (FBS)<br>KerSFM medium<br>Show 2 more
bacterial culture/bacterial culture/bacterial species/ bacterial species							
name*	Aggregatibacter actinomycetemcomitans<br>Streptococcus oralis	methicillin resistant Staphylococcus aureus	Staphylococcus aureus<br>Streptococcus oralis	Staphylococcus aureus<br>Staphylococcus epidermis	Staphylococcus aureus	Pseudomonas stutzeri<br>Staphylococcus simulans	Actinomyces naeslundii<br>Porphyromonas gingivalis<br>Streptococcus oralis<br>Veillonella dispar
culture medium ingredient*	yeast extract<br>yeast extract<br>yeast extract<br>yeast extract<br>Brain heart infusion (BHI)<br>Todd-Hewitt broth<br>Tryptone Soya Broth<br>yeast extract<br>yeast extract	Muller-Hinto-broth	Todd-Hewitt broth<br>Tryptone Soya Broth	Luria Bertani broth<br>Luria Bertani broth<br>Luria Bertani broth<br>Luria Bertani broth<br>Luria Bertani broth<br>Luria Bertani broth	Luria Bertani broth	CASO Bouillon<br>CASO Bouillon<br>CASO Bouillon<br>CASO Bouillon<br>CASO Bouillon<br>CASO Bouillon	Brain heart infusion (BHI)<br>Brain heart infusion (BHI)<br>Brain heart infusion (BHI)<br>Vitamin K<br>Brain heart infusion (BHI)<br>Brain heart infusion (BHI)<br>Brain heart infusion (BHI)<br>Vitamin K<br>Brain heart infusion (BHI)<br>Brain heart infusion (BHI)<br>Brain heart infusion (BHI)<br>Vitamin K<br>Brain heart infusion (BHI)<br>Brain heart infusion (BHI)<br>Brain heart infusion (BHI)<br>Vitamin K<br>Brain heart infusion (BHI)<br>[illegible]

Neele Brümmer, Katharina Doll-Nikutta, Patrik Schadzek, Carina Mikolai, Andreas Kampmann, Dagmar Wirth, Andrea Hoffmann, Philipp-Cornelius Pott, Meike Stiesch, Oliver Karras, and Sören Auer. (2025). 3D In Vitro Models for Implant-Associated Infections [Data set]. Open Research Knowledge Graph. https://doi.org/10.48366/R1368153
