# Supplementary figures and images for: Better models, better treatment? a systematic review of current three dimensional (3D) in vitro models for implant-associated infections
Source: Front Bioeng Biotechnol. 2025 Apr 25;13:1569211. doi: 10.3389/fbioe.2025.1569211 (PMC12061920; doi:10.3389/fbioe.2025.1569211)

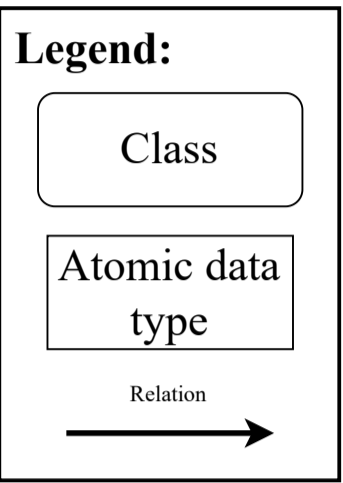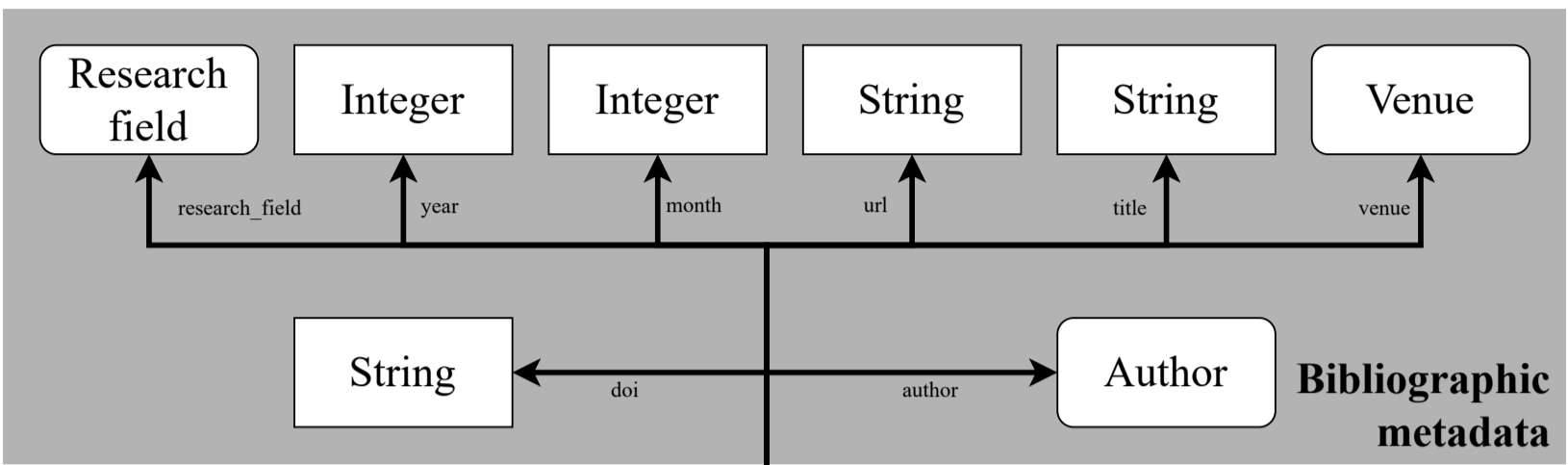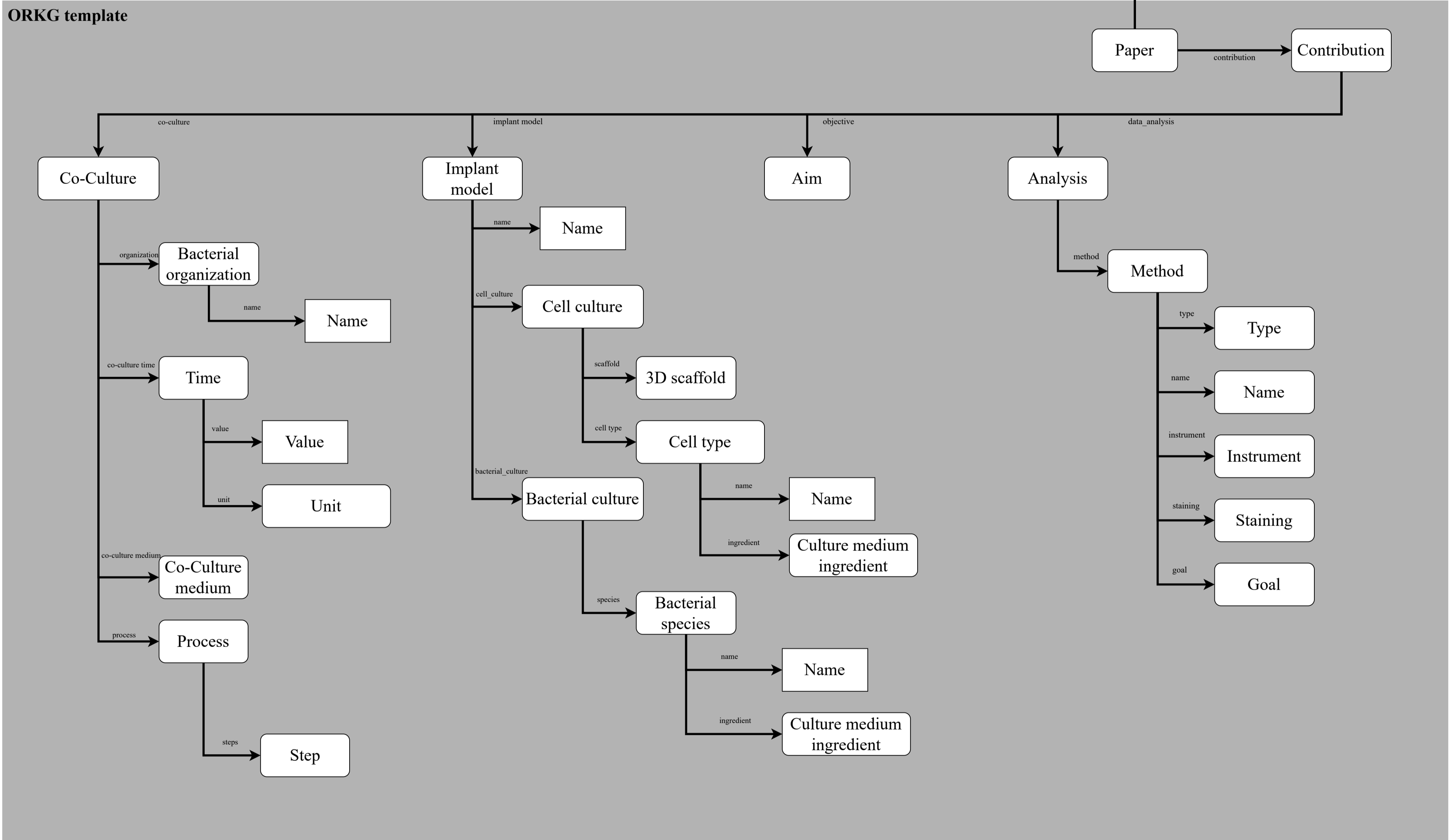

Supplement: Supplementary file 3 [file DataSheet1.pdf]
